# A chemically defined system supports two distinct types of stem cell from a single blastocyst and their self‐assembly to generate blastoid

**DOI:** 10.1111/cpr.13396

**Published:** 2023-01-02

**Authors:** Baojiang Wu, Zhiqing Yang, Yijie Liu, Jianwen Li, Chen Chen, Xihe Li, Siqin Bao

**Affiliations:** ^1^ The State Key Laboratory of Reproductive Regulation and Breeding of Grassland Livestock Inner Mongolia University Hohhot China; ^2^ Research Centre for Animal Genetic Resources of Mongolia Plateau College of Life Sciences, Inner Mongolia University Hohhot China; ^3^ Inner Mongolia Saikexing Institute of Breeding and Reproductive Biotechnology in Domestic Animal Hohhot China

## Abstract

The pluripotent stem cells exist in a narrow window during early development and its derivation depends on intrinsic and extrinsic growth signalling in vitro. It has remained challenging to derive two or three distinct cell lines that are representative of blastocyst‐stage lineages from one preimplantation embryo simultaneously in a chemical defined condition. Therefore, it is desirable to establish a system by manipulating extrinsic signalling in culture to derive multiple types of stem cells from a single blastocyst. This study used a defined medium containing Activin A, WNT activator and LIF (ACL medium), enabling establishment of ACL‐ESCs and ACL‐XEN cells from one blastocyst. ACL‐blastoids were generated by suspending ACL‐ESCs and ACL‐XEN cells with ACL‐blastoid medium in three‐dimensional culture system. Lineage markers expression of ACL‐blastoids were performed by immunofluorescence. Our results indicate that ACL‐ESCs and ACL‐XEN cells derived from one blastocyst represent ICM and PrE lineages. Importantly, we obtained ACL‐blastoid from ACL‐ESCs and ACL‐XEN cells self‐aggregation, partially recapitulating early development and initiation of early implantation events. This study would not only provide ACL culture system for derivation and maintenance of two types of cell lines corresponding to ICM as well as PrE, but also reconstruct blastoids with them to deepen our understanding of early embryogenesis and widen insights into translational application of stem cells.

## Graphical abstract

ACL medium enabling to establishment of ACL‐ESCs and ACL‐XEN cells from one blastocyst. Importantly, obtained ACL‐blastoid from ACL‐ESCs and ACL‐XEN cells self‐aggregation, partially recapitulating early development and initiation of early implantation events. This research not only provide ACL culture system for derivation and maintenance of two types of cell lines corresponding to ICM as well as PrE, but also reconstruct blastoids with them to deepen our understanding of early embryogenesis and widen insights into translational application of stem cells.

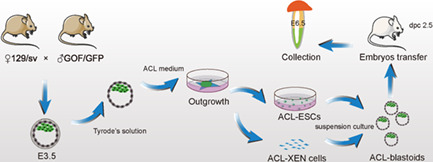

## 1 INTRODUCTION

During the early stage of embryogenesis in mammals, the developmental potential of cells in the embryo is gradually restricted by a series of cleavages and early differentiation. During morula compaction, it is an initial cell fate commitment that outer cells segregate from the blastomeres as trophectoderm (TE), which surrounds the inner cell mass (ICM). Accordingly, a blastocyst is formed following cavitation. Prior to implantation, the inner cell mass generates epiblast progenitor cells and the bipotent extra‐embryonic primitive endoderm (PrE), which differentiates into the endodermal lineages, parietal endoderm (PE) and visceral endoderm (VE).^1^, ^2^, ^3^

Three representative stem cells derived from blastocysts can self‐renew, maintain and proliferate in vitro as ESCs,^4^, ^5^, ^6^ TSCs^7^, ^8^ and XEN cells,^9^ respectively. ESCs derive and maintain self‐renewal and pluripotency properties via the addition of two small‐molecule inhibitors of glycogen synthase kinase 3 and mitogen‐activated protein kinase (2i), and leukaemia inhibitory factor (LIF).^6^ Only ESCs have been confirmed through the gold‐standard germline transmission test, and can result in the generation all body tissues.^10^ TSCs are derived from trophoblasts and maintained in vitro in the presence of FGF4 and Heparin, while retaining their ability to differentiate into multiple cell types of the placenta.^7^, ^11^, ^12^ XEN cells can be established and continuously passaged using the same TSC culture conditions or serum‐containing medium, contributing to the extraembryonic endoderm cell types.^13^, ^14^

All three cell lineages are maintained indefinitely in their particular cell culture system as stable cell lines with essential signal requirements. The trophectoderm is specified due to compaction during morula and is restricted in its developmental potential with the expression of genes related to trophectoderm, which is distinct from pluripotent ESCs, the corresponding cell lineages of ICM in vitro. However, ICM^15^ and ESCs^16^ still can partially differentiate into trophoblast lineages. The trophectoderm can be induced via the ectopic expression of the caudal‐type homeobox transcription factor 2 (Cdx2) in ESCs. The overexpression of Gata4 or Gata6 in ESCs is sufficient to induce the establishment of self‐renewing XEN cells, which are the in vitro counterparts of the PrE lineage of the mouse embryo.^17^, ^18^ In fact, XEN‐like cells have been found to exist within ESCs cultured on XEN protocol.^4^, ^19^ Human or mouse XEN cells can be derived from ESCs via the addition of the endoderm agonist, Activin and WNT, and expand in the presence of LIF and low insulin without the requirement for gene manipulation.^20^, ^21^ Similarly, retinoic acid (RA) and Activin A applied progressively to ESCs can promote the differentiation of XEN cells in mice.^22^ Although TSCs colonies are present during XEN cell line derivation from ICM or single blastocysts under TSCs derivation conditions, XEN cells remain as the dominant group.^7^, ^9^ Altogether, all three blastocyst lineages are not completely separated, but communicate with each other via several conserved signalling pathways. As representative cell lines of blastocyst lineages, ESCs, TSCs and XEN cells were recently combined with each other to model the in vivo interactions between embryonic and extra‐embryonic lineages were used exclusively to reconstruct embryo‐like structures. Early attempts to generate embryo‐like structures via the spontaneous differentiation of ESCs in suspension culture resulted in disorganized cell aggregates termed as embryoid bodies.^19^, ^23^ The reconstruction of embryo‐like structures in vitro offers new opportunities for understanding embryogenesis. Based on previous studies, ESCs and TSCs cooperate in vitro to form structures that resemble embryonic day 3.5 (E3.5) blastocysts, termed blastoids.^24^ Furthermore, three types of stem cells, ESCs, XEN cells and TSCs aggregate together, forming gastrulating embryo‐like structures (or gastruloids).^25^, ^26^ Recently, Sozen et al. demonstrated the generation of blastocyst‐like structures from mouse extended pluripotent stem cells (EPSC) aggregated with TSCs that contained three spatially segregated lineages representative of the Epi, TE and PrE.^27^ As the three types of stem cells were derived from different culture media, identifying the factors that play an important role in blastoids proved difficult. The EPSCs could generate blastoids; however, the culture medium of EPS‐blastoids was complicated, comprising FGF4, Heparin and serum.^28^

Several studies have shown that human blastocyst‐like structures can be generated from human pluripotent stem cells^29^, ^30^ or induced pluripotent stem cells (iPSC) from the reprogramming of fibroblasts.^28^ Although embryo‐like structures recapitulate the key features of early embryonic development, they do not support the development of bona fide embryos. In addition, ESCs can develop into organoids, including gastrulating embryo‐like structures, which present an invaluable system to recapitulate early development in vitro.^31^, ^32^, ^33^, ^34^, ^35^, ^36^, ^37^, ^38^, ^39^ Based on previous studies, stem cell cultures can self‐assemble in vitro to generate gastruloids in human.^31^ Most morphological characteristics and gene expression levels of gastruloids are very similar to those of natural embryos, with differences, such as the lack of gene expression associated with extra‐embryonic cell types.^36^ The developmental defect of stem cell‐derived blastoids or gastruloids is mainly associated with the in vitro culture conditions; compared with in vivo development, it is difficult to provide the full requirements for stem cell‐derived blastoids or gastruloids under in vitro culture conditions.

Different signalling pathways play an important role in development and control differentiation into particular germ layers in vitro, such as Nodal/Activin,^40^, ^41^, ^42^, ^43^, ^44^, ^45^, ^46^ WNT,^47^, ^48^, ^49^, ^50^, ^51^, ^52^, ^53^, ^54^ BMP^55^, ^56^, ^57^ and LIF‐STAT^58^, ^59^ signalling pathways, which are not only critical for cell lineage derivation and maintenance, but also for the generation of embryo‐like structures in vitro. In mice, the activation of TGF‐β signalling via the Activin A pathway was previously found to support ESCs proliferation,^60^ but is also essential for PrE promotion when combined with CHIR99021 (CHIR), the stimulator of Wnt signalling, similar to that for human ESCs differentiation.^20^, ^21^ CHIR is not only a Wnt/β‐catenin agonist, in fact, CHIR represses GSK‐3 to promote naive pluripotency and reduced variability within cohorts of cells.^6^ During pre‐implantation development, the LIF‐STAT pathway plays a critical role in blocking Epi differentiation, supporting PrE expansion, the maintenance of XEN cells in vitro. Accordingly, XEN cells derived and proliferated in the condition of EMFIs or EMFI‐CM, providing an adequate source of LIF.^9^ FGF/ERK signalling is required for TE differentiation through FGFR1 and is important for PrE specification. Thus, FGF/ERK signalling plays a key role in maintaining appropriate proportions of cell types in the blastocyst.^61^ Recently, studies have been conducted on the induction of embryo‐like structures by modulating several signalling pathways. Bone morphogenetic protein (BMP4), Nodal, STAT, MAPK and WNT signalling are critical for blastoid formation and trophoblast epithelial morphogenesis.^24^ Although the WNT signalling pathway is important for blastocyst development,^54^ the combination of the WNT and FGF4 signalling pathways efficiently reconstruct EPSC‐blastoids.^28^ Nodal/Activin signalling can also support the development of the extra‐embryonic compartment in gastruloid and natural embryos in early post‐implantation stages.^62^ To date, the derivation and maintenance of these three types of stem cell lines, which can be employed as the origins of embryo‐like structures under the same culture conditions, have not been reported. As all the necessary requirements are available, three types of stem cell lines can be derived from one blastocyst using the same culture conditions.

Due to restricted accessibility to human embryonic specimens donated for research and ethical limitations, mouse blastocysts were used in this study.^63^ By focusing on Activin A, WNT activator (CHIR99021) and the LIF signalling pathway, we tested the following hypothesis: there is a defined culture condition sufficient to govern two or three stem cell induction and maintenance. Through medium test experiments, we found that the addition of Activin A, CHIR99021 and LIF (ACL medium) supported the establishment of two types of stem cells, ESCs (ACL‐ESCs) and XEN‐like (ACL‐XEN) cells, from one blastocyst. The transcriptional characteristics and developmental competence indicated that ACL‐ESCs and ACL‐XEN cells were equivalent to the inner cell mass and primitive endodermal lineages of the pre‐implantation embryo. Notably, ACL‐ESCs and ACL‐XEN cells were demonstrated to self‐organize into blastocyst‐like ACL‐blastoids.

## 2 MATERIALS AND METHODS

### 2.1 Animals

Oct4‐△PE‐GFP (GOF/GFP) transgenic mice^64^ used in this study were strains with a mixed background of MF1, 129/sv and C57BL/6J. Sterile‐male bred, pseudopregnant mice for chimera production were strains from CD1 (ICR).

### 2.2 Derivation of ACL‐ESCs

Mouse blastocysts (E3.5) were isolated from 129/sv females mated with GOF/GFP transgenic males, whose green fluorescence indicated expression in the ICM of blastocysts, primordial germ cells (PGC) in vivo, and ESCs.^64^ Blastocysts were collected at the relevant stages from the uterus in M2 medium (Sigma‐Aldrich). The zona pellucida were then removed using Acidic Tyrode's Solution (Sigma‐Aldrich), and blastocysts were washed three times with M2. The embryos were then placed in a 24‐well fibronectin‐coated (16.7 μg/ml, Millipore) plate, with ACL medium consisting of N2B27 medium supplemented with Activin A (20 ng/ml, R&D Systems), CHIR9902 (3 μM, Miltenyi Biotech) and leukaemia inhibitory factor (1000 IU/ml, Millipore). N2B27 medium was used as the basic medium, which included a 1:1 mixture of DMEM/F12 medium (Gibco), neurobasal medium (Gibco), 0.5% N2 (Gibco), 1% B27 (Gibco), 1% Glutamax (Gibco), 1% NEAA (Gibco), 100 μM β‐mercaptoethanol (Sigma) and 1% penicillin/streptomycin (Gibco). Within the following 3–5 days, most of the blastocysts attached and then formed heterogeneous outgrowths, which were domed and GOF/GFP positive in the middle. In contrast, the “flat” epithelial‐like and GOF/GFP negative cells were surrounding in a radial flow. On day 7–9, when the diameter of GOF/GFP positive clone grew to around 200 μm, the colony was picked, cut into small pieces with glass needle for 2–3 passages, and dissociated with Accutase (Gibco). Thereafter, domed GOF/GFP positive clones resembling ground‐state ESCs in morphology were formed. Here, these cells were called ACL‐ESCs.

### 2.3 Derivation of ACL‐XEN cells

The “flat” and GOF/GFP negative outgrowths that spread out on day 8–10 was disaggregated into single cells with TrypLE (Gibco) and cultured in ACL medium equally. When the colonies reached 80–90% confluence, cells were passaged every 2–3 days in a 24‐well fibronectin‐coated plate. These cells, referred to as ACL‐XEN cells, could perform self‐renewal for over 35 passages.

### 2.4 Derivation of 2i/L‐ESCs

E3.5 blastocysts with GOF/GFP collected from the uterus and zona pellucidae were removed using Acidic Tyrode's Solution (Sigma‐Aldrich) and placed in a 24‐well plate coated with fibronectin (16.7 μg/ml, Millipore) at least 0.5 h before use. Serum‐free ESC culture medium (2i/L) was prepared as previously described.^6^ Briefly, N2B27 basic medium was supplemented with PD0325901 (PD, 1 μM, Miltenyi Biotech), CHIR99021 (CH, 3 μM, Miltenyi Biotech), and leukaemia inhibitory factor (LIF, 1000 IU/ml, Millipore); the medium was named 2i/L‐medium. Within 5–6 days, the ICM of the blastocyst cultures grew efficiently, and outgrowth occurred. When colonies grew to around 200 μm in diameter, they were picked and minced into pieces using glass needles and cultured in 2i/L medium. Colonies were passaged manually for 2–3 passages as described above, and then treated with Accutase (Gibco) every 2 days. Finally, these cells were named 2i/L‐ESCs.

### 2.5 Derivation of XEN cell lines

To derive the XEN cell lines, a previous protocol^13^ with modifications was employed. E3.5 embryos with a CD1 (ICR) background were placed in Tyrode's solution (Sigma‐Aldrich) to remove the zona pellucida, washed three times with M2 (Sigma‐Aldrich), and transferred into XEN cell derivation medium (30% TS medium and 70% Feeder‐conditioned medium from mouse embryonic fibroblasts) without any growth factors. The plates were pre‐treated with fibronectin 0.5 h before use. The blastocyst attached and formed an outgrowth following 3–5 days of culture; the medium was changed every 3 days. On days 9–12, the spreading outgrowth was disaggregated into single cells with TrypLE for 7 min; the dissociation was terminated using XEN cell derivation medium. The cell pellets were resuspended and seeded onto 24‐well plates pre‐treated with fibronectin. After 2–3 passages at high confluence, XEN cells grew efficiently.

### 2.6 Immunofluorescence (IF) staining

To conduct immunofluorescence for 2D (Dimensions) cell culture, ACL‐blastoids, early mouse embryos, chimeric embryos and post‐implantation embryo‐like structures, the samples were briefly washed with DPBS (Gibco) and fixed with freshly prepared 4% paraformaldehyde (Solarbio) in DPBS for 15 min at room temperature. The samples were then permeabilised for 30 min with 1% BSA (Gibco) and 0.1% Triton X‐100 (Sigma) in DPBS, and incubated with primary antibodies diluted in the buffer described above at 4 °C overnight. After three washes with 1% BSA and 0.1% Triton X‐100 in DPBS for 5 min each, the samples were incubated with fluorescence‐conjugated secondary antibodies diluted in the above buffer for 1 h (2D cell culture, ACL‐blastoids, chimeric embryos and early mouse embryos) or overnight (post‐implantation embryo‐like structures) at room temperature in dark. The samples were mounted in Vectashield with DAPI (Vector Laboratories) after one wash with DPBS containing 1% BSA and 0.1% Triton X‐100, and two washes with DPBS. Images were acquired using a Nikon confocal microscope (Nikon, Tokyo, Japan) or a Zeiss LSM 710 confocal microscope. The primary antibodies and dilutions were as follows: mouse monoclonal OCT4 (BD Biosciences, 1:200), rat monoclonal NANOG (eBioscience, 1:500), goat polyclonal SOX2 (Santa Cruz, 1:200), rat monoclonal E‐cadherin (Takara, 1:200), goat polyclonal GATA4 (R&D Systems, 1:100), goat polyclonal GATA6 (R&D Systems, 1:100), goat polyclonal SOX17 (R&D Systems, 1:200), mouse monoclonal CDX2 (Biogenex, 1:200), rabbit monoclonal YAP (Abcam, 1:500), rabbit polyclonal N‐cadherin (Abcam, Cambridge, 1:200), and rat polyclonal Podocalyxin (PCX) (R&D Systems, 1:500). All secondary antibodies were Alexa Fluor 488 or Alexa Fluor 568 (Molecular Probes, Eugene, CA).

### 2.7 Production of chimeras from ACL‐ESCs/ACL‐XEN cells

Using a piezo‐assisted micromanipulator attached to an inverted microscope, approximately 8–12 ACL‐ESCs were injected into eight‐cell embryos collected from E2.5 CD1 (ICR) mice recipient, which were then cultured in KSOM medium (Millipore) overnight at 37 °C in a 5% CO_2_ atmosphere to obtain chimeric blastocysts. After re‐expansion of the blastocoel cavity, some chimeric blastocysts were transferred to the uteri of pseudopregnant CD1 (ICR) mice at 2.5 days post coitus (dpc) to generate chimeras, and the remaining chimeric blastocysts were cultured for another 24 h and fixed in 4% paraformaldehyde for immunofluorescence. Full‐term chimeras were confirmed by the coat colour pattern of the pups at birth.

To generate chimeric embryos of ACL‐XEN cells, 8–12 cells were gently injected into the perivitelline space of eight‐cell embryos. After 48 h of culture in KSOM medium at 37 °C in a 5% CO_2_ atmosphere, chimeric embryos were hatched and then fixed for IF. Embryonic day 6.5 (E6.5) CD1 (ICR) wild‐type gastrulae were also collected, and ACL‐XEN cells were microinjected into the cavity of gastrulae, which were cultured in ACL medium for 48 h to trace ACL‐XEN cells.

### 2.8 Generation of ACL‐blastoids

Following incubation with Accutase and TrypLE, ACL‐ESCs with GOF/GFP and ACL‐XEN cells with tdTomato were dissociated into single cells, and mixed at a ratio of 1:5 with a total concentration of 1 × 10^5^ cells/ml. After centrifugation for 3 min at 1300 rpm, the cell pellet was resuspended in 800 μl ACL‐blastoid medium and transferred into a well of a 24‐well plate coated with Anti‐Adherence Rinsing Solution (StemCell Technologies) at least 0.5 h before use, which was day 0 of the entire aggregation process. The ACL‐blastoid medium contained 50% N2B27 medium and 50% KSOM medium as a basic medium supplemented with Activin A (20 ng/ml, R&D Systems), CHIR9902 (3 μM, Miltenyi Biotech) and LIF (1000 IU/ml, Millipore). From day 2, 400 μl of the supernatant was carefully replaced with fresh medium every 2 days. In the first 2 days, no cavity formation occurred; however, small aggregates were observed. The emergence of ACL‐blastoids was observed at day 3 after cell seeding, and ACL‐blastoids continued to increase, reaching a normal size, resembling E3.5 blastocyst around day 5. The ACL‐blastoids were retrieved with a mouth pipette and fixed for IF or RNA extraction.

### 2.9 Derivation of three types of stem cells from ACL‐blastoids

For ESCs derivation, individual ACL‐blastoids were transferred onto a fibronectin‐coated 24‐well plate and cultured in 2i/L medium, which comprised N2B27 basic medium with PD0325901 (1 μM, Miltenyi Biotec), CHIR99021 (3 μM, Miltenyi Biotec) and LIF (1000 IU/ml, Millipore). Within 3–4 days, ACL‐blastoids attached and formed outgrowths. As individual colonies grew to around 200 μm in diameter, they were minced into pieces and transferred into a new 24‐well plate with fibronectin pretreatment. Colonies grown for 3–4 days were treated with Accutase for cell line derivation.

To derive the TSC, ACL‐blastoid were transferred to one well of a 24‐well plate with a layer of irradiated MEF feeders^7^ and outgrowth was observed within 3–4 days. On day 6–7, the outgrowth was retrieved, dissociated with 0.05% trypsin–EDTA (Biological Industries), and plated into a new 24‐well plate with MEF feeders. TSCs were cultured in TSCs basal medium composed of RPMI 1640 (Gibco) supplemented with 20% foetal bovine serum (FBS) (Gibco), 1× GlutaMAX (Gibco), 1× Sodium pyruvate (Gibco), and 0.1 mM 2‐mercaptoethanol (Sigma), 25 ng/ml recombinant human FGF4 (rhFGF4) (R&D Systems) and 1 μg/ml Heparin (Sigma‐Aldrich).

XEN cell lines were established according to a previous protocol,^13^ with modifications. ACL‐blastoids were plated individually in a 24‐well plate pre‐coated with fibronectin in XEN derivation medium (30% TS medium and 70% feeder‐conditioned medium from mouse embryonic fibroblasts) and supplemented with 25 ng/ml rhFGF4 and 1 mg/ml Heparin. An outgrowth was observed around day 3. In the following days, the medium was changed every 3 days. At around day 10, the XEN cells were dissociated into single cells using TrypLE for 7 min at 37 °C. Dissociation was stopped with release medium, and the cells were collected via centrifugation at 1300 rpm for 3 min. Thereafter, the pellets were resuspended and plated into a 24‐well plate pre‐coated with fibronectin. FGF4 and Heparin were removed from the XEN derivation medium after 2–3 stable passages, once the XEN cells were established and grew well.

### 2.10 Embryos and ACL‐blastoids transfer

The 2.5 dpc recipient was anaesthetised with Avertin (Aibei), and the uterine horn was exposed via surgery. Chimeric embryos or ACL‐blastoids on day 5 were picked and transferred into KSOM droplets using a mouth pipette and washed three times. Subsequently, approximately 10–15 chimeric embryos or ACL‐blastoids were transferred to each side of the uterine horn and pre‐punctured with a needle. At 6.5 or 7.5 dpc, post‐implantation embryo‐like structures were collected from deciduae at implantation sites in the dissected uterus.

### 2.11 Karyotype

Both ACL‐ESCs and ACL‐XEN cells were prepared for cytogenetic analysis via treatment with colchicine (Sigma) at a final concentration of 0.2 μg/ml for 2.5 h to accumulate cells in metaphase. Following cell harvesting, the cell pellets were exposed to 8 ml 0.075 M KCl (Sigma) for 10 min at 37 °C for hypotonic treatment, and cold fixative solution prepared with 1 ml of methanol: acetic acid (3:1) was gently added and mixed. After discarding the supernatant, the cells were fixed three times at 37 °C with 8 ml fixative solution for 30 min each. Thereafter, the cells were suspended in 0.5 ml cold fixative solution and dropped onto pre‐cold clean slides, which were dried for 1 h at 70 °C in an incubator. After cooling to 25 °C approximately, the slides were stained with Giemsa (Sigma) for 10 min, and the unfixed dyes were removed via washing with distilled water. The slides were photographed using a microscope (Nikon), and the preparations were analysed using LUCIA Cytogenetics (Lucia).

### 2.12 RT‐qPCR

Total RNA was extracted from cultured cells using RNeasy Mini Kit (Qiagen). ACL‐blastoids or natural blastocysts were isolated and purified using the PicoPure RNA Isolation Kit (Thermo Fisher Scientific) following the manufacturer's instructions. Complementary DNA (cDNA) was synthesized using a Reverse Transcription System (Promega). Real‐time quantitative polymerase chain reaction (RT‐qPCR) was performed using the SYBR FAST Universal qPCR kit (KAPA) on a LightCycler 96 Instrument II (Roche). Each experiment was performed in triplicate. The primer pairs are listed in Table S1.

### 2.13 RNA extraction and sequencing

Total RNA was extracted from approximately 2 × 10^6^ cells using Trizol reagent kit (Invitrogen) according to the manufacturer's recommendations. mRNA was enriched by Oligo(dT) Beads from total RNA, which was then fragmented into short fragments using fragmentation buffer, and transcripts were reversed into cDNA with random primers. Second‐strand cDNA were synthesized by DNA polymerase I, RNase H, dNTP and buffer. Then the cDNA fragments were purified with QiaQuick PCR extraction kit (Qiagen), end repaired, poly(A) added, and ligated to Illumina sequencing adapters. The ligation products were size selected by agarose gel electrophoresis, PCR amplified and sequenced using Illumina HiSeq2500 by Gene Denovo Biotechnology Co. (Guangzhou, China).

### 2.14 RNA‐seq and analysis

Before alignment, raw data were trimmed to remove reads with more than 10% low‐quality bases and trimmed adaptors. Thereafter, the clean reads were mapped to the mouse reference genome (10 mm) using TopHat (2.0.12) with default settings.^65^ HTSeq (0.6.1) was used for reads counting, and the RefSeq gene expression level was estimated using the reads per kilobase transcriptome per million reads (RPKM) method. In vivo data for mouse embryos E4.5 Epi (E‐MTAB‐2958),^66^ E4.5 PrE,^66^ ESCs (GSE119985),^60^ TSCs^67^ and XEN cells (GSE159181)^67^ were downloaded and processed identically. DEGs in different samples were determined using the edgeR package, with a fold change ≥2 and *p* ≤ 0.5.^68^ Unsupervised hierarchical clustering (UHC) analysis was performed using the R *hclust* function. t‐SNE was performed using the *Rtsne* function. Heatmaps of the selected genes were generated using the R *heatmap.2* function. Principal component analysis was performed using the R *prcomp* function. Gene Ontology (GO) analysis was performed using Metascape (http://metascape.org). Trend analysis of DEGs was performed using Short Time‐series Expression Miner software.^69^

### 2.15 Statistical analyses

Statistical analyses were performed using the GraphPad Prism software (v8.0.2). Data are presented as mean *±* SD. Significance between each group was measured using unpaired two‐tailed Student's *t* test, and a value of *p* < 0.05 was considered statistically significant.

## 3 RESULTS

### 3.1 Activin A replaces the MEK inhibitor for the derivation of ESCs from blastocyst

**FIGURE 1:**
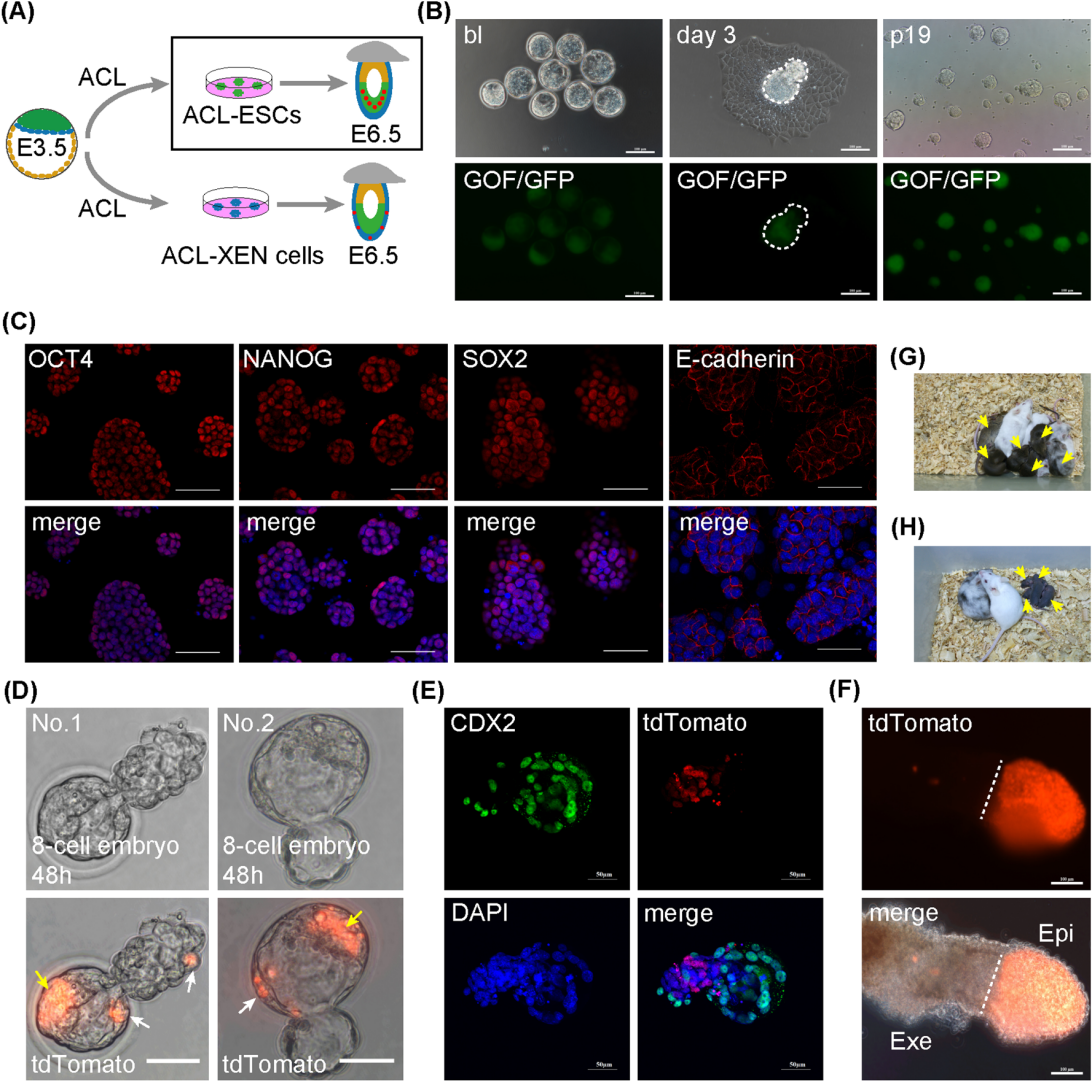
Activin A replaces MEK inhibitor to support ESCs pluripotency. (A) Schematic of ACL‐ESCs derivation from E3.5 blastocysts. (B) Derivation of ACL‐ESCs lines from blastocysts. bl, blastocyst. Scale bars, 100 μm. (C) IF staining assays of OCT4, SOX2, NANOG, E‐cadherin in ACL‐ESCs. DAPI stained the nucleus. Scale bars, 50 μm. (D) Localization of ACL‐ESCs (tdTomato+) in ICM contribution (yellow arrows) and trophectoderm contribution (white arrows) in chimeric embryos in vitro cultured for 48 h. Scale bars, 50 μm. (E) The 30 E4.5 blastocysts (hatching) developing from 8‐cell embryos injected with ACL‐ESCs were stained for CDX2 (green), which indicated no merge with H2B tdTomato+ donor cells. DAPI stained the nucleus. Scale bars, 50 μm. (F) E6.5 chimeras generated by ACL‐ESCs (tdTomato+). Scale bars, 100 μm. Exe, extra‐embryonic ectoderm; Epi, epiblast. (G) Chimeric pups (yellow arrows) generated by injecting ACL‐ESCs into ICR host blastocysts (*n* = 3 independent experiments). (H) F1 Pups (yellow arrows) generated by ACL‐ESCs derived chimera male mated with ICR female.

Early embryonic development is affected by different signalling pathways at different stages of development; however, the development of all embryonic cell lineages occurs in the same environment of the oviducts or uterus. Therefore, under certain conditions, different stem cell lines may be generated from a single blastocyst. In ESCs culture medium, 2i/L (MEK inhibitor PD0325901, GSK3 inhibitor CHIR99021 and LIF), the MEK inhibitor, PD0325901, prevents ESCs differentiation in long‐term culture.^6^ Nodal/Activin signalling is critical to embryonic development, particularly for extra‐embryonic development. Here, Activin A was used to replace the MEK inhibitor (PD0325901) in 2i/L medium (Activin A, CHIR99021 and LIF), which resulted in the ACL medium, to establish new embryonic stem cells from blastocysts. Oct4‐△PE‐GFP (GOF/GFP, mixed background of MF1, 129/sv and C57BL/6J strains) 129/sv F1 mouse blastocysts were directly placed in chemically defined ACL medium on fibronectin‐coated cell culture plates (Figure 1A). Two types of cells were discovered in ACL medium: GOF/GFP‐positive ESC‐like cells and GOF/GFP‐negative flat cells after 3 days of culture (Figures 1B and S1A). GOF/GFP‐positive cells were picked and digested with Accutase in the subsequent passages; these GOF/GFP‐positive cells were defined as ACL‐ESCs. ACL‐ESCs were morphologically similar to ESCs and maintained self‐renewal capability over passage 19 (p19) (Figure 1B).

The key features of ACL‐ESCs relative to those of ESCs were determined. ACL‐ESCs exhibited high alkaline phosphatase (AP) activity (Figure S1B) and a normal karyotype (Figure S1C). IF staining revealed that ACL‐ESCs expressed pluripotency‐related proteins, OCT4, NANOG, SOX2 and E‐cadherin (Figure 1C). To determine the in vivo developmental potency of ACL‐ESCs, we injected H2B tdTomato‐labelled ACL‐ESCs into eight‐cell embryos and evaluated their chimeric development in vitro. tdTomato‐positive ACL‐ESCs were found to contribute robustly to the ICM (73/73, 100%) and a small number of cells to trophectoderm (TE) (46/73, 63%) at 48 h post‐injection (Figures 1D and S1D). However, ACL‐ESCs at the TE position were negative for the trophectoderm marker, CDX2 (Figures 1E and S1D). We proceeded to determine their ability to contribute to chimeric embryos at E6.5. Based on our findings, ACL‐ESCs successfully developed into epiblasts (19/19, 100%) (Figures 1F and S1E). Notably, full‐term chimeras (17/26, 65%) were obtained from ACL‐ESCs and their germline transmission abilities were verified (Figures 1G, H and S1F). To further determine the differentiation ability of ACL‐ESCs in vitro, 2i/L‐ESCs and ACL‐ESCs were cultured in N2B27 basic medium without any components. After 3 days of in vitro differentiation, RT‐qPCR analysis was performed. Compared with 2i/L‐ESCs, the relative expression of all three germ layer markers was significantly increased in ACL‐ESCs, except *Hand1* (Figure S1G). These results indicate that ACL‐ESCs have a strong differentiation ability that depends on environmental changes. Taken together, these data suggest that Activin A replaces the MEK inhibitor to support derivation of ACL‐ESCs from blastocysts, and ACL‐ESCs present similar pluripotency features to ESCs and have stronger differentiation ability than ESCs in vitro.

### 3.2 ACL culture condition supports XEN‐like cells derivation from blastocyst

**FIGURE 2:**
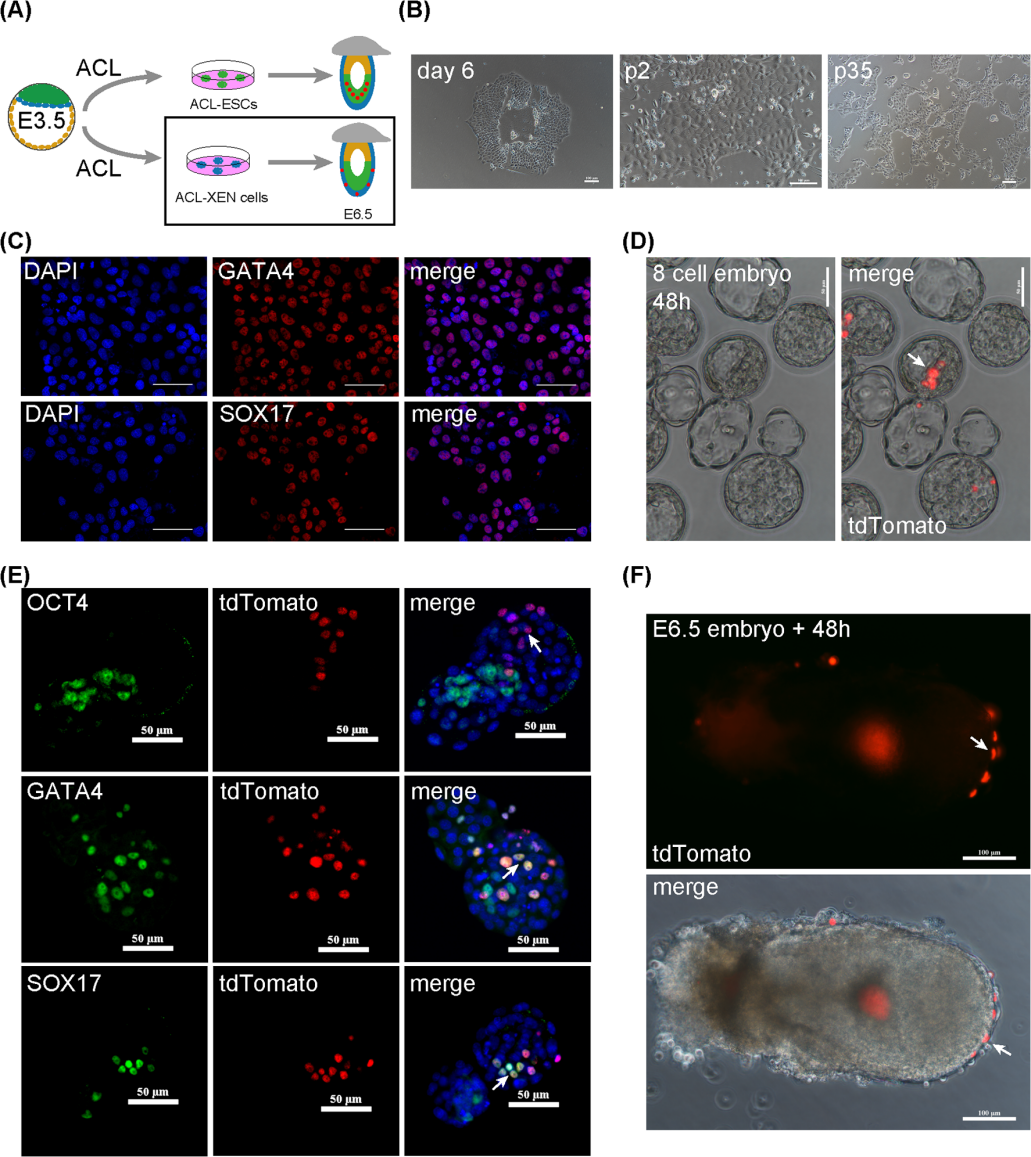
ACL supports XEN‐like cells derivation from blastocyst. (A) Schematic of ACL‐XEN cells derivation from E3.5 blastocyst. (B) Representative images of derivation of ACL‐XEN cells at day 6, and treated with TrypLE for passage 2 (p2) and passage 35 (p35). Scale bars, 100 μm. (C) IF staining assays of GATA4 and SOX17 in ACL‐XEN cells. DAPI stained the nucleus. Scale bars, 50 μm. (D) Bright‐filed images of E4.5 chimeric embryos generated after 8‐cell embryos injection of ACL‐XEN cells (tdTomato+) and cultured for 48 h in vitro. ACL‐XEN cells (tdTomato+) in primitive endoderm (write arrow). Scale bars, 50 μm. (E) IF staining of OCT4, GATA4 and SOX17 (green) of E4.5 chimeric embryos (*n* = 30) generated after 8‐cell embryos injection of ACL‐XEN cells (tdTomato+) and cultured for 48 h in vitro. Arrows indicate ACL‐XEN cells contribute to PrE. DAPI stained the nucleus. Scale bars, 50 μm. (F) Bright filed and fluorescent images of E6.5 embryos cultured for 48 h in vitro, after ACL‐XEN cells (tdTomato+) injected. Arrows indicate H2B tdTomato+ cells in the Visceral endoderm. Scale bars, 50 μm.

When blastocysts were placed in ACL medium for 3–5 days, GOF/GFP‐positive cells were picked, and GOF/GFP‐negative flat cells were observed (Figure 2A). GOF/GFP‐negative flat cells propagated rapidly, were highly refractile, and appeared epithelial‐like, with features coinciding with the typical morphology of XEN cells or TSCs^7^, ^9^, ^13^, ^14^ (Figure 2B). These cells displayed genome stability after more than 35 passages (Figures 2B and S2A) and highly expressed primitive endoderm cell lineage‐related genes, such as *Gata4*, *Gata6* and *Sox17* (Figure S2B). The IF staining results also showed that high levels of GATA4 and SOX17 proteins were observed in ACL cultured epithelial‐like cells (Figure 2C), and not detected CDX2 positive trophectoderm‐like cells (Figure S2C). Therefore, GOF/GFP‐negative flat cells were designated as ACL‐XEN cells. Previous work suggests that XEN cells become dominant over trophoblast from blastocyst outgrowths.^9^ Therefore, we investigated whether TS‐like cells existed in early stage of ACL cells derivation. We cultured blastocysts in ACL medium do not pick up the GOF/GFP‐positive cells, and mixed with GOF/GFP‐negative flat cells. We performed immunofluorescence staining, and found ACL cultured cells in early stage (passage 6) expressed pluripotent protein maker OCT4, SOX2 and endoderm protein maker GATA4, however, no trophectoderm marker CDX2 was detected (Figure S2D, E). We proved that the majority cells are ES‐like cells and XEN‐like cells in ACL culture system. The above results are consistent with the previous report.

XEN cells can self‐renew in in vitro culture and differentiate into PrE derivatives, such as VE and PE, and contribute to chimeras (in vivo) in a lineage‐appropriate manner, highlighting the developmental potential of their origin.^9^ To determine the developmental potential of ACL‐XEN cells in vivo, ACL‐XEN cells with H2B tdTomato‐labelled were injected into eight‐cell embryos, which were then cultured for 48 h. The result showed ACL‐XEN cells displayed a significant contribution to PrE (18/30, 60%) (Figures 2D and S2F), whereas 40% of injected embryos did not find ACL‐XEN cells, may occur apoptosis after injection in vitro culture. tdTomato positive ACL‐XEN cells also co‐expressed with GATA4 and SOX17 in chimeras (Figure 2E), as well as not detected OCT4 and CDX2 positive ACL‐XEN cells (tdTomato+) (Figures 2E and S2G). Thus, ACL‐XEN cells have no ability to contribute to the ICM/TE. Finally, to confirm whether ACL‐XEN cells contribute to the VE in vivo, ACL‐XEN cells with tdTomato‐labelled were introduced into gastrulae at E6.5, following cultured in ACL medium for 48 h. Interestingly, tdTomato‐positive cells could migrate to the visceral endoderm after 48 h of culture (Figure 2F). The above results indicate that ACL conditions support the generation of ACL‐ESCs and ACL‐XEN cells from one blastocyst, with features resembling those of ESCs and XEN cells. Notably, the convenient protocols for deriving XEN‐like cells described here can be completed within 2–3 weeks and without the use of serum‐containing and MEF‐conditioned medium.^13^, ^14^ Hence, chemically defined ACL conditions may open new avenues for XEN cell research and help deepen our understanding of an in vitro microenvironment for the development of multiple cell lineages.

### 3.3 Global transcriptional features of ACL‐ESCs and ACL‐XEN cells

**FIGURE 3:**
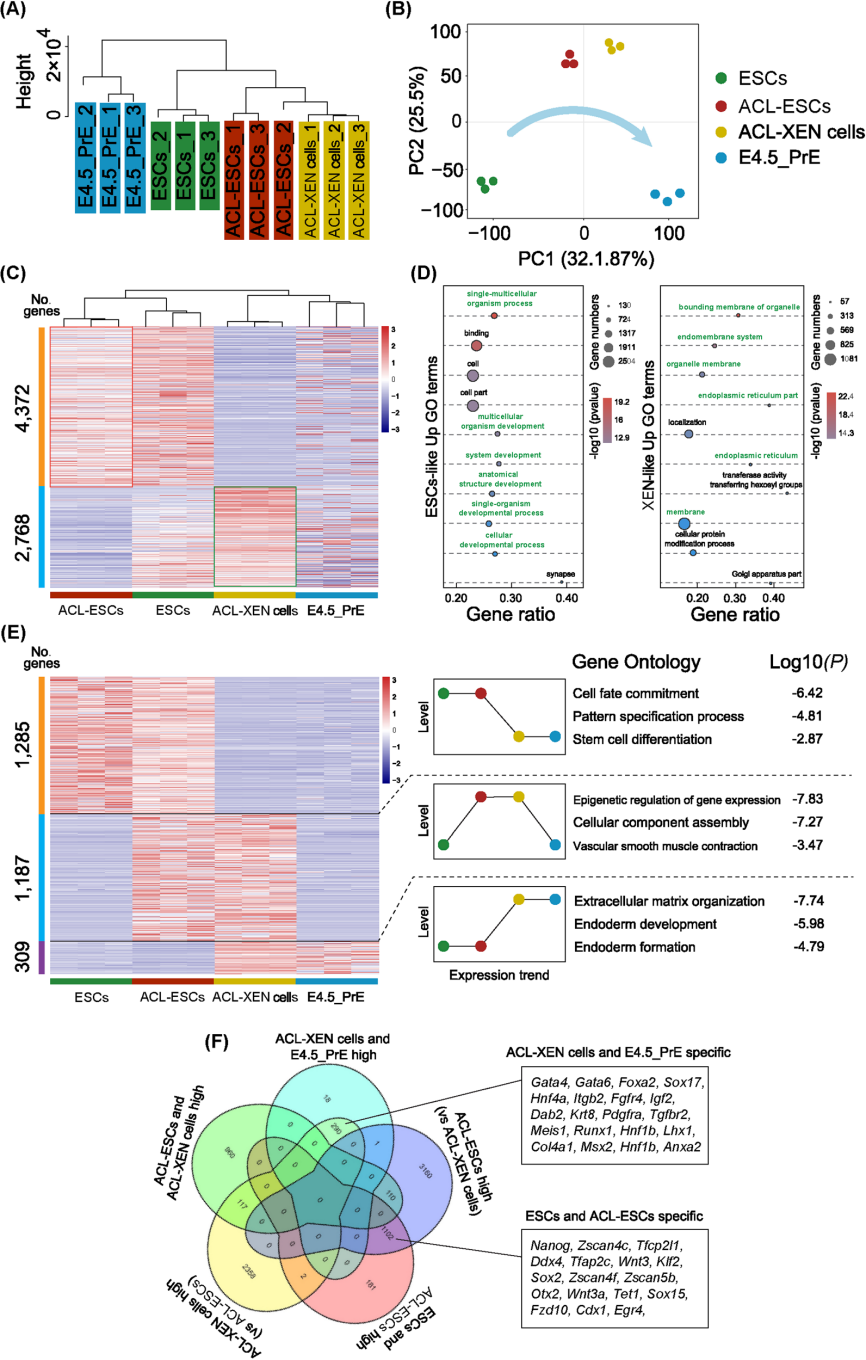
Analyses of molecular features of ACL‐ESCs and ACL‐XEN cells. (A) Unsupervised hierarchical clustering (UHC) of whole‐genome transcriptome on three biological replicates of three types of stem cell lines and E4.5_PrE (primitive endoderm). (B) t‐SNE analysis of gene expression of three types of stem cell lines and E4.5_PrE. Arrow indicates that ACL‐ESCs and ACL‐XEN are developmentally intermediate between ESCs and E4.5_PrE. (C) Heatmap showing scaled expression values of a total of 7140 differentially expressed genes (mean log2(normalized read counts) > 2, log2(fold change) > 2, adjusted p value <0.05) in ACL‐ESCs and ACL‐XEN cells, and compared with ESCs and E4.5_PrE. (D) The top representative GO terms (biological process) for ACL‐ESCs and ACL‐XEN cells upregulated genes. (E) Comparison of ACL‐ESCs, ACL‐XEN cells, ESCs and E4.5_PrE. Among differentially expressed genes, a total of 1285 genes (top) were significantly highly expressed in ACL‐ESCs and ESCs compared with ACL‐XEN cells and E4.5_PrE; a total of 1187 genes (middle) were significantly upregulated in ACL‐ESCs and ACL‐XEN cells compared with ESCs and E4.5_PrE; a total of 309 genes (bottom) were significantly upregulated in ACL‐XEN cells and E4.5_PrE compared with ESCs and ACL‐XEN cells (*n* = 3 biological replicates on four groups). (F) Venn diagram showing overlap of specific genes among ACL‐ESCs, ACL‐XEN cells, ESCs and E4.5_PrE.

To determine whether ACL‐ESCs and ACL‐XEN cells have distinct molecular features, RNA sequencing was performed on the dynamics of ACL‐ESCs and ACL‐XEN cells and compared with those of ESCs^60^ and E4.5‐PrE,^66^ respectively. Unsupervised hierarchical clustering (UHC) revealed that ACL‐ESCs were close to ACL‐XEN cells (Figure 3A), supporting the two cell lines derived from the same culture condition. t‐SNE analysis showed that ACL‐ESCs and ACL‐XEN cells were intermediate between ESCs and E4.5‐PrE (Figure 3B). A total of 7140 genes were differentially expressed in ACL‐ESCs compared with ACL‐XEN cells, among which 4372 genes were upregulated in ACL‐ESCs (Figure 3C). Notably, the 4372 genes were also upregulated in ESCs (Figure 3C). Based on GO analysis, the upregulated genes were associated with multicellular organism development, system development and cellular developmental processes (Figure 3D). Another 2768 genes were upregulated in ACL‐XEN cells, and the GO terms were associated with the bounding membrane of organelle, endomembrane system and organelle membrane (Figure 3D). These transcriptional profiles show that the gene expression patterns of ACL‐ESCs and ACL‐XEN cells were distinct from each other, and were intermediate between ESCs and E4.5‐PrE, respectively.

To further investigate the molecular features of ACL cultured cells, We compared ACL‐ESCs and ACL‐XEN cells with ESCs, TSCs,^67^ XEN cells,^67^ E4.5‐Epi^66^ and E4.5_PrE. UHC analysis demonstrated that ACL‐ESCs were close to ESCs (Figure S3A), in line with the IF staining and in vivo chimera assays results. We also found that ACL‐XEN cells were clustered closely to XEN cells and intermediate between E4.5 and TSCs (Figure S3A, B). To determine the characteristic of ACL‐ESCs and ACL‐XEN cells, trophectoderm‐ and endoderm‐related makers were evaluated. The heatmap shows that trophectoderm‐related makers (*Cdx2*, *Gata2*, *Tead4*, etc.) were highly expressed in TSCs, however, endoderm‐related makers (*Gata4*, *Sox17*, *Foxa2*, etc.) were found significantly up‐regulated in both ACL‐XEN cells and XEN cells (Figure S3C). The molecular characteristics of ACL‐XEN cells were consistent with the IF staining results that endoderm makers such as SOX17 and GATA4 were high‐expressed, but expression of trophectoderm maker CDX2 was negative in ACL‐XEN cells. Above results indicated that ACL‐XEN cells may intermediate between PrE and TE, but the precise regulatory mechanism still needs to be further investigated.

In addition, we used the short time‐series expression miner (STEM) method^69^ to analyse the gene expression profiles of ESCs, ACL‐ESCs, ACL‐XEN cells and E4.5‐PrE. Interestingly, 1285 differentially expressed genes were significantly highly expressed in ACL‐ESCs and ESCs compared with those in ACL‐XEN cells and E4.5‐PrE (Figure 3E). GO terms were associated with ESC pluripotency, such as cell fate commitment, pattern specification process and stem cell differentiation (Figure 3E). Notably, 309 genes were significantly upregulated in ACL‐XEN cells and E4.5‐PrE compared with ACL‐ESCs and ESCs (Figure 3E). GO terms were associated with the features of XEN cells, such as extracellular matrix organization, endoderm development and endoderm formation (Figure 3E). Meanwhile, a total of 1187 genes were highly expressed in both ACL‐ESCs and ACL‐XEN cells (Figure 3E); these genes may serve as the ACL condition related target genes, and are associated with the epigenetic regulation of gene expression, cellular component assembly, and vascular smooth muscle contraction processes (Figure 3E). However, the functional role of ACL target genes in ACL‐ESCs and ACL‐XEN cell self‐renewal remains to be determined. Moreover, as shown in the Venn diagram, ACL‐ESCs were closer to ESCs, and ACL‐XEN cells were closer to E4.5‐PrE (Figure 3F). Taken together, these data strongly suggest that ACL conditions can establish and maintain two types of stem cell lines derived from a single blastocyst.

### 3.4 Blastocyst‐like structures reconstructed from ACL‐ESCs and ACL‐XEN cells

**FIGURE 4:**
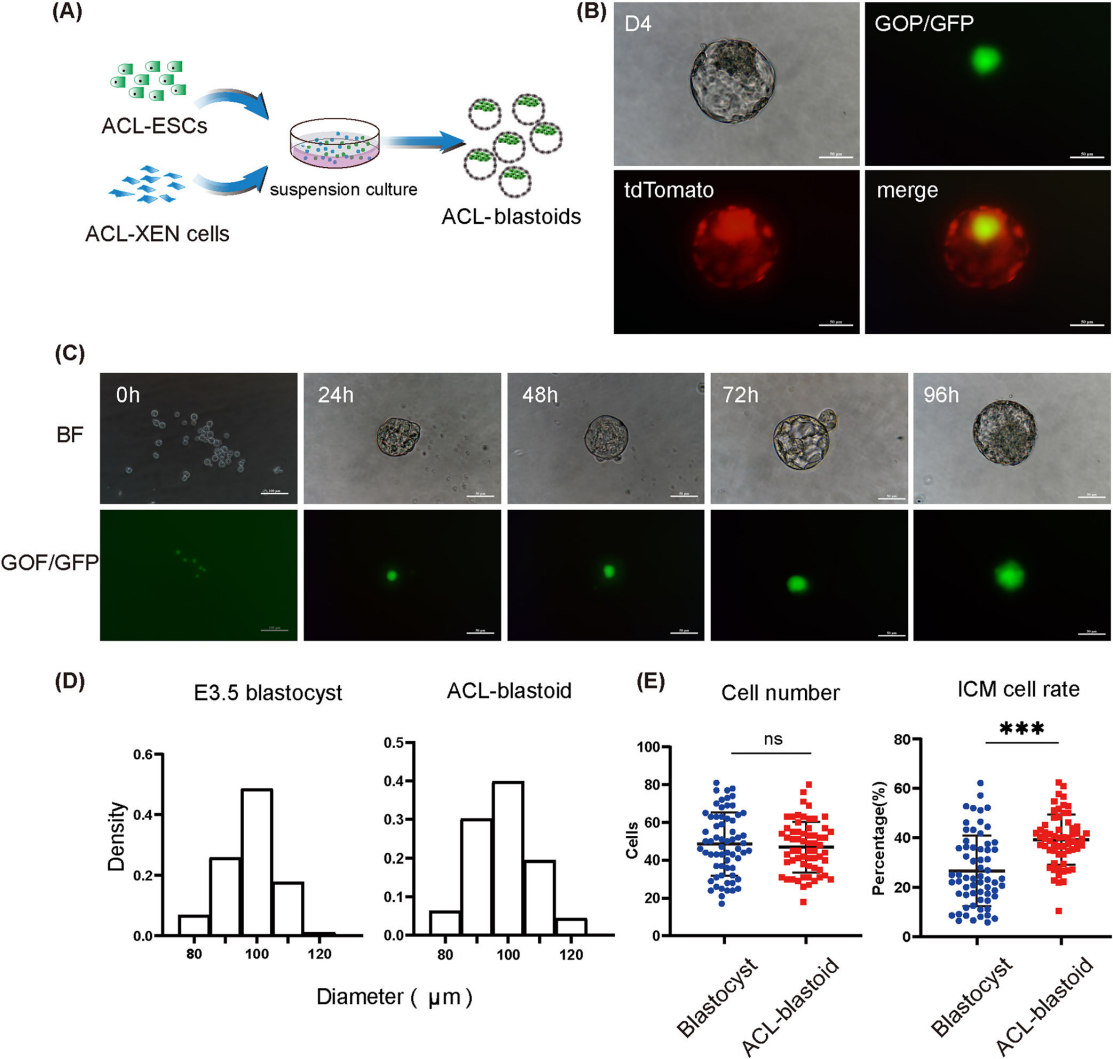
Generation of blastoid from ACL‐ESCs and ACL‐XEN cells. (A) Diagram of self‐assembly into ACL‐blastoids with ACL‐ESCs and ACL‐XEN cells. (B) Bright‐field, fluorescent images and a merged live image of an ACL‐blastoid at day 4 with ACL‐ESCs (GOF/GFP+) and ACL‐XEN cells (tdTomato+). Scale bar, 100 μm. (C) Representative bright‐field (top) and fluorescence images (bottom) of the formation process of ACL‐blastoids at the indicated time point. Scale bar, 100 μm (0 h), 50 μm (24–96 h). BF, bright field. (D) Histograms showing the distribution of diameter of 175 E3.5 blastocysts (left) and 206 ACL‐blastoids (right). (E) Total cell number and ICM cell ratio were quantified between 64 E3.5 blastocysts and 62 ACL‐blastoids. Data are means ± SD, ***p < 0.0001.

After fertilization, cleavage stage embryonic cells communicate with each other and develop into blastocysts containing three different cell types (ICM, PrE and TE).^2^ We proceeded to determine whether the two types of stem cells derived from one blastocyst (ACL‐ESCs and ACL‐XEN cells) were cultured together under ACL conditions and could aggregate into blastocyst‐like structures (blastoids) (Figure 4A). To confirm this hypothesis, ACL‐ESCs and ACL‐XEN cell lines with reporters (GOF/GFP and H2B tdTomato, respectively) were used to trace their spatial locations for aggregation. When ACL‐ESCs and ACL‐XEN cells were co‐cultured on FN‐coated plates with ACL medium, there were some small clones in which GOF/GFP‐positive ACL‐ESCs were surrounded by epithelial‐like cells developed from ACL‐XEN cells (Figure S4A). We examined the expression of the key proteins, OCT4 in ACL‐ESCs and SOX17 in ACL‐XEN cells, and found that both proteins could be detected in co‐cultured cells (Figure S4B). Interestingly, some cells co‐expressed OCT4 and SOX17 proteins (Figure S4B), indicating that the co‐culture system could drive ACL‐ESCs to develop further into epiblast and XEN cells. This result is consistent with ICM resulting in the epiblast and extra‐embryonic primitive endoderm.^2^

Under the adherent culture conditions, the co‐cultured cells experienced difficulty forming blastoid structures. Thus, we attempted to use suspension culture conditions, treated cell culture plates with anti‐adherence solution,^28^ and aggregated two types of stem cells using different cell proportions based on recent reports, in which the suitable cell‐type proportion and cell–cell communication were highlighted to play an important role in cell proliferation and development.^70^ Initially, we aggregated ACL‐ESCs and ACL‐XEN cells at ratios of 1:1, 1:5 and 1:10, respectively, and found that mixing ACL‐ESCs with ACL‐XEN cells at a 1:5 ratio successfully induced cavity formation in a small number of cell aggregates, whereas most aggregations failed to form blastoids at ratios of 1:1 and 1:10 (Figures 4B and S4C). Using the above optimized conditions, we consistently observed the development of ACL‐blastoids (Figure 4C) and found that they continued to enlarge and reach an early blastocyst‐like size at around day 4. We also counted the total number of ACL‐blastoids, solid and vacuole structures, respectively, and found that formation efficiency of ACL‐blastoids with typical blastocyst‐like morphology were 68.42% at day 4 (Figure S4D). Notably, the average diameter and total cell number of ACL‐blastoids were comparable to those of E3.5 blastocysts, and the ICM cell number of ACL‐blastoids was higher than that of E3.5 blastocysts (Figure 4D, E). We examined whether ACL‐ESCs and ACL‐XEN cell have the capacity to produce blastoids or gastruloids in ETX medium.^25^ Most of the cells in ETX medium were found to be differentiated or apoptotic, and only few cells could induce smaller aggregations, but failed to form cavities of blastoids or gastruloids in morphology (Figure S4E). Collectively, our results demonstrate that ACL‐blastoids were reconstructed from ACL‐ESCs and ACL‐XEN cell aggregates in ACL medium.

### 3.5 ACL‐blastoids resemble blastocysts in cell lineage allocation

**FIGURE 5:**
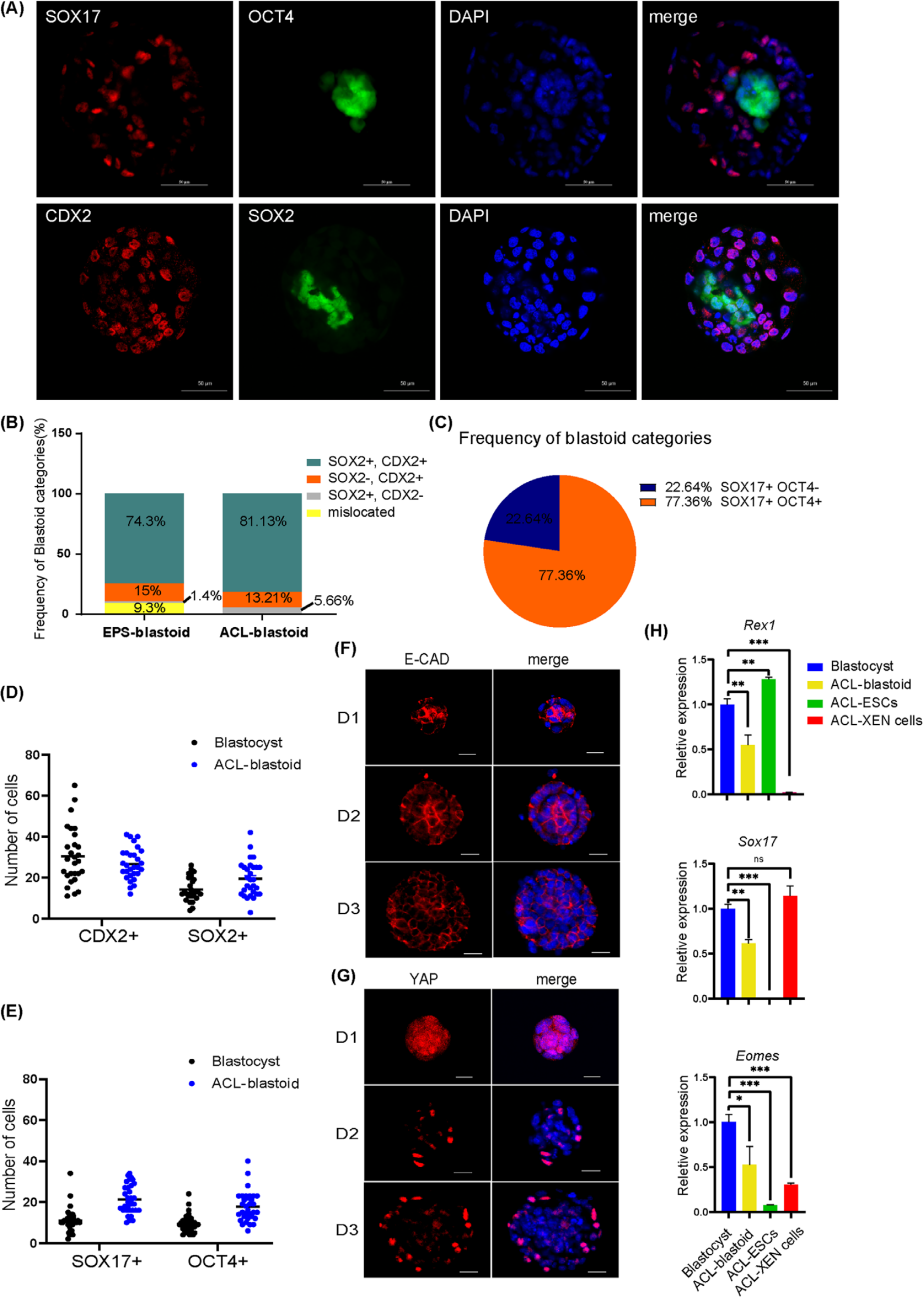
Lineage potency of ACL‐blastoids. (A) IF staining of OCT4, SOX2, SOX17 and CDX2 in ACL‐blastoids cultured for 5 days. All ACL‐blastoids below were collected at day 5. DAPI stained the nucleus. Scale bars, 50 μm. (B) The frequency of ACL‐blastoid categories based on the expression of CDX2 and SOX2 in the chart. *n* = 53 ACL‐blastoids, compared with previous published EPS‐blastoid. (C) The frequency of 53 ACL‐blastoid categories based on the expression of SOX17 and OCT4 in the chart. (D) Quantification of the cells number with SOX2+ or CDX2+ in 29 blastocysts and 28 ACL‐blastoids. (E) Quantification of the cells number with OCT4+ or SOX17+ in 36 blastocysts and 33 ACL‐blastoids. (F) IF staining of E‐cadherin (E‐CAD) in early stage of ACL aggregates at the indicated time point. DAPI stained the nucleus. Scale bars, 20 μm. (G) IF staining of Active‐YAP (YAP) in early stage of ACL aggregates at the indicated time point. DAPI stained the nucleus. Scale bars, 20 μm. (H) Relative expression of three blastocyst lineages genes (*Rex1*, *Sox17* and *Eomes*) measured by qPCR in individual blastocyst at day 4, ACL‐blastoid at day 5, ACL‐ESCs, ACL‐XEN cells. Blastocyst was used as control. Error bars indicate means ± SD (*n* = 3). *p* values were calculated by unpaired two‐tailed Student's *t* test, *p* < 0.05.

To determine whether the ACL‐blastoids develop into three blastocyst lineages, ICM, TE and PrE, as natural E3.5 mouse blastocysts, immunofluorescence was employed. The ACL‐blastoids collected on day 5 had a morphology similar to that of the E3.5 blastocysts. Cells inside the ACL‐blastoid expressed the pluripotency factors, OCT4 and SOX2, while the cells on the outer layer of the ACL‐blastoid expressed the trophectoderm marker, CDX2 (Figure 5A). SOX17 positive PrE‐like cells were also detected to surround the OCT4 positive compartment; however, some of the observed SOX17 positive cells were mislocated in ACL‐blastoids (Figure 5A). Approximately 81.1% of ACL‐blastoids were SOX2 and CDX2 positive, and approximately 13.2% and 5.7% of ACL‐blastoids were only CDX2 or SOX2 positive, respectively (Figure 5B). These results are comparable to those reported previously for EPS‐blastoids.^28^ Approximately 77.4% of ACL‐blastoids were SOX17 and OCT4 positive, and approximately 22.6% of ACL‐blastoids were only SOX17 positive (Figure 5C). The number of ICM, TE and PrE‐like cells in ACL‐blastoids (collected on day 5) was counted based on OCT4, SOX2, SOX17 and CDX2 immunostaining. The number of OCT4, SOX2 and SOX17 positive cells increased, while that of CDX2 positive cells decreased in blastoids compared with E3.5 blastocysts (Figures 5D, E and S5A). We proceeded to determine whether ACL‐ESCs and ACL‐XEN cells are important for generating blastoids and used 2i/L‐ESCs to replace ACL‐ESCs and aggregate with ACL‐XEN cells in ACL suspension conditions. We observed blastocyst‐like structures with some GOF/GFP‐positive cells inside and surrounded with SOX17 positive cells, but with no CDX2 positive cells (Figure S5B, C). Furthermore, we examined the potential of blastoid formation by changing ACL‐XEN cells to traditional XEN cells (labelled with tdTomato) and performing aggregation with ACL‐ESCs and 2i/L‐ESCs, respectively. No blastocyst‐like structures were obtained from these two combinations (Figure S5D). These results indicate that both ACL‐ESCs and ACL‐XEN cells are necessary for the generation of ACL‐blastoids.

Finally, we determined whether key cellular and molecular events that feature early pre‐implantation development could be recapitulated during ACL‐blastoid formation. We traced the dynamics of the two types of cell aggregation during the first 3 days. On days 1 and 2, the cells started to form compact aggregates, and the cell adhesion protein, E‐cadherin, began to accumulate at the cell–cell junctions, similar to compacted embryos (Figure 5F). Importantly, blastocyst cavity‐like structures started to form on day 3 (Figure 5F). In mouse early embryogenesis, TE and ICM lineages are specified from the early blastocyst stage, and YAP signalling is critical for this process.^8^, ^71^, ^72^, ^73^, ^74^ On day 2, YAP was found in some outside cells of ACL‐ESCs and ACL‐XEN cell aggregates; on day 3, the nucleus of most outside cells expressed the active YAP protein (Figure 5G). The molecular characteristics of ACL‐blastoids (day 5) were verified via qPCR using a single ACL‐blastoid compared with a single blastocyst. Some markers of blastocyst lineage expression levels were found to be similar to those of natural blastocysts, such as *Rex1*, *Sox17* and *Eomes* (Figure 5H); however, some of the lineage markers, *Oct4*, *Nanog*, *Gata6*, *Cdx2*, *Gata2* and *Gata3*, were lower in the ACL‐blastoids than the natural blastocysts (Figure S5E). Together, these findings indicate that ACL‐blastoids resemble blastocysts, with inner cells expressing pluripotency and outer cells expressing trophectoderm lineage markers.

### 3.6 In vitro and in vivo developmental potential of ACL‐blastoids

During embryonic development, early blastomeres specialize in the ICM and TE, and the ICM results in the PrE and epiblast of the blastocysts. Moreover, ESCs, TSCs and XEN cells can be directly derived from blastocysts.^2^, ^75^ We determined whether ACL‐blastoids could also lead to these three stem cell lines. Here, we successfully generated ESCs lines from ACL‐blastoids under 2i/L culture condition. The morphologies of ACL‐blastoid‐derived ESCs were similar to those of natural blastocysts derived from ESCs and expressed the pluripotency protein, SOX2 (Figure S5F). TSCs lines were also derived from ACL‐blastoids using FGF4‐ and Heparin‐containing conditioned medium on feeder cells and expressed the TSCs key factor CDX2 (Figure S5F). XEN cell lines were also successfully established from ACL‐blastoids and expressed the PrE transcription factor, GATA4 (Figure S5F).

**FIGURE 6:**
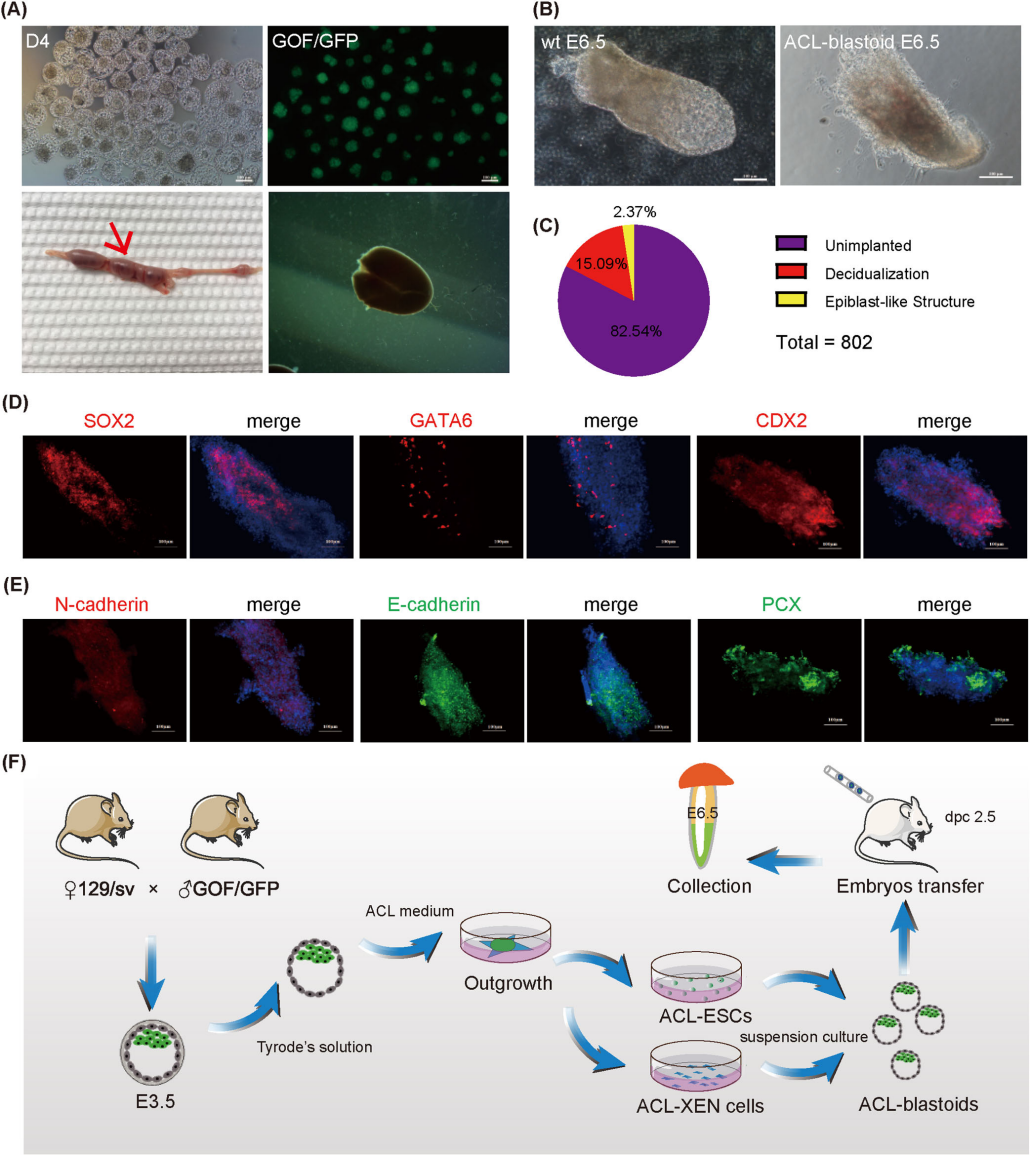
In vivo developmental potential of ACL‐blastoids. (A) The formation of E6.5 decidua in the mouse uterus after ACL‐blastoids with GOF/GFP (top) transferred to 2.5 dpc recipient. Red arrow indicates deciduae. Scale bars, 100 μm. (B) Left: wild type E6.5 gastrula. Right: in vivo ACL‐blastoid developed to gastrulation‐like structure and collected from deciduae at 6.5 dpc embryos. Scale bars, 100 μm. (C) A pie chart showing the frequency of implantation capacity of ACL‐blastoids. *n* = 802. (D) IF staining of SOX2, GATA6 and CDX2 in tissue sections of in vivo ACL‐blastoid developed to gastrulation‐like structures and collected from deciduae at 7.5 dpc. DAPI stained the nucleus. Scale bars, 50 μm. (E) IF staining of N‐cadherin, E‐cadherin and PCX in tissue sections of in vivo ACL‐blastoid developed to gastrulation‐like structures and collected from deciduae at 7.5 dpc. PCX, podocalyxin. DAPI stained the nucleus. Scale bars, 50 μm. (F) Overview of generating ACL‐blastoids with ACL‐ESCs and ACL‐XEN cells derived from one blastocyst, and followed by in vivo developmental potential of ACL‐blastoid.

We assessed the in vivo developmental potential by transferring ACL‐blastoids into pseudopregnant mice at 2.5 and 3.5 dpc (Figure 6A). At 6.5–7.5 dpc, we collected developmental ACL‐blastoids and identified the formation of decidua in the uteri of surrogate ACL‐blastoids (Figure 6A). Overall, approximately 17% of the transferred ACL‐blastoids were implanted and induced decidualization (Figure 6A, B). However, only approximately 2% of the transferred ACL‐blastoids formed gastrulation‐like structures (Figure 6C). Although ACL‐blastoids can successfully form gastrulation‐like structures, the size of gastrulation‐like structures varied and was markedly smaller than that of the control (Figure 6B). Importantly, IF results indicated that deciduae induced by gastrulation‐like structures contained SOX2, GATA6 and CDX2 positive cells (Figure 6D). Meanwhile, lumenogenesis in post‐implantation embryos is regulated by podocalyxin (PCX).^76^ The levels of PCX and cell adhesion proteins, E‐cadherin and N‐cadherin, were also detected in the deciduae derived from ACL‐blastoids (Figure 6E), whereas the distribution patterns of PCX, E‐cadherin and N‐cadherin were disorganized in the deciduae derived from ACL‐blastoids. Whether artificial embryos have developmental abilities resembling those of natural embryos remains to be further examined. In summary, ACL‐blastoids led to the generation of ESCs, TSCs and XENs in vitro and developed into gastrulation‐like structures in vivo (Figure 6F).

## 4 DISCUSSION

Mouse pluripotent stem cells were established in different media with overlapping components. According to Ying et al., the inhibition of Mek1/2 (PD) and Gsk3b (CHIR), and the activation of STAT3/LIF signalling, known as 2i/L medium, enhance the derivation of ESCs and promote ground‐state pluripotency.^6^ The establishment of epiblast‐derived stem cells (EpiSCs), a pluripotent cell type, requires the presence of fibroblast growth factor (FGF) and Activin A^77^ and the derivation of TSCs and XEN cells depends on Activin/Nodal and FGF signalling.^9^, ^22^, ^78^ Moreover, PrE can be induced from naïve ESCs, and gastrulation‐stage definitive endoderm (DE) from EpiSCs when CHIR cooperates with Activin A.^20^ A previous study revealed that prolonged Mek1/2 suppression impairs the chromosomal stability of ESCs.^79^ The cytokine, LIF, which potently promotes mouse ESC identity, is a key component of ESCs culture conditions,^80^, ^81^ while the LIF‐STAT pathway may play a role in XEN cell maintenance with the expression of some components, such as LIFR, gp130, JAK1, JAK2, STAT1 and STAT3.^9^ Our findings revealed that LIF alone is sufficient to maintain ESCs pluripotency in a hypermethylated state.^82^ Considering the above results, we concluded that Activin/Nodal, CHIR and LIF (ACL medium) are sufficient to derive two distinct stem cell lines from blastocysts. However, how cell lineages of blastocysts develop in ACL medium and precisely regulate mechanisms for stem cell self‐renewal remain largely elusive.

Based on our findings, ACL‐ESCs and ACL‐XEN cells are derived from one blastocyst and retain a global transcriptome signature of the inner cell mass and PrE of blastocysts, respectively. Accordingly, ACL‐ESCs express key factors specific to naïve pluripotency and efficiently contribute to chimeric development and germline transmission, which is characteristic of naïve pluripotency. ACL‐XEN cells are marked by primitive endoderm‐specific markers, such as *Gata4*, *Gata6* and *Sox17*, and chimerism experiments provide direct evidence for the contribution of the embryonic endoderm in embryos at E6.5. Such defined culture condition of ACL allows us to re‐establish and examine the coordinated interactions of ESCs, TSCs and XEN cells from one blastocyst, which have remained inaccessible until now.

Recent studies have shown that blastoids represent an accessible, scalable, and tractable model system that will be valuable for many applications in basic research and translational approaches.^24^, ^27^, ^28^, ^29^, ^30^, ^83^, ^84^ The blastoids were generated from three different stem cells (ESCs, TSCs and XEN cells) with high efficiency and developed into a gastrula‐like stage.^27^, ^28^, ^84^ These three types of stem cells originate from blastocysts and depend on different signalling pathway regulators under in vitro culture conditions.^75^ Natural blastocysts develop in the environment of the oviducts or uterus, and three lineages always exist in the same developing environment. The findings of this study demonstrated that two distinct types of stem cell lines could be derived from one blastocyst and reconstructed into blastoids under the same culture conditions.

Here, we demonstrate that ACL‐ESCs and ACL‐XEN cells can be reconstituted to blastocyst‐like structures, which recapitulate early embryonic developmental processes and initiate early implantation events. Although we successfully generated ACL‐blastoids from ACL‐ESCs and ACL‐XEN cell aggregation, the levels of most of the three lineage markers were lower than those in natural blastocysts, especially TE lineages, and consistent with previous reports that TE markers are lower in stem cell‐derived embryonic models.^85^ Nodal/Activin signalling is required for TSCs renewal in culture and blastoid induction in previous reports.^78^, ^86^, ^87^ Our culture conditions comprise Activin A, and we hypothesized ACL‐ESCs aggregating with ACL‐XEN cells might initiate TSCs differentiation and form blastocyst‐like structures. We attempted to generate blastoids by aggregating 2i/L‐ESCs and ACL‐XEN cells in ACL medium, but failed to obtain the TE marker, CDX2 positive cells. Overall, ACL‐ESCs were demonstrated to be necessary for ACL‐blastoid formation. Further, the development of CDX2 positive cells was found to rely on the interaction between the ACL‐ESCs and ACL‐XEN cells; however, whether ACL‐ESCs directly differentiate into TE cells, or push ACL‐XEN cells to reverse to TE cells remains unclear and should be further explored.

Here, we described a modified cell culture system that generates two types of stem cell lines from one blastocyst. These cells were used to reconstruct ACL‐blastoids, which had many similarities to blastocysts at the morphological, developmental, molecular and functional levels; however, these processes were imperfect and perhaps less neatly regulated than those in natural embryos. Overall, a simple approach to derive two types of stem cells from one blastocyst and generate ACL‐blastoids is to capture the mechanism of embryo lineage segregation.

## AUTHOR CONTRIBUTIONS

Baojiang Wu, Xihe Li and Siqin Bao conceived the study; Baojiang Wu and Zhiqing Yang derived the ACL‐ESCs/ACL‐XEN cells and performed the in vivo embryo experiment; Zhiqing Yang generated and analysed the molecular properties of ACL‐blastoids. Zhiqing Yang, Yijie Liu, Jianwen Li and Chen Chen analysed the molecular properties of ACL‐ESCs/ACL‐XEN cells. Baojiang Wu, Zhiqing Yang and Siqin Bao wrote the manuscript with inputs from all authors.

## CONFLICT OF INTEREST

The authors declare that they have no competing interests.

## ETHICS STATEMENT

All animal experiments were performed using humane methods in compliance with the animal ethical standards approved by the Animal Research Committee of Inner Mongolia University, China.

## Supporting information


**DATA S1.** Supporting Information

## Data Availability

RNA‐seq data were deposited in the Genome Sequence Archive (GSA) under the accession number, CRA005808. All data that support the conclusions of this study are available from the authors upon reasonable request.
