# Supplementary material for: A chemically defined system supports two distinct types of stem cell from a single blastocyst and their self‐assembly to generate blastoid
Source: Cell Prolif. 2023 Jan 2;56(6):e13396. doi: 10.1111/cpr.13396 (PMC10280139; doi:10.1111/cpr.13396)
Supplement: Supplementary file 1 — DATA S1. Supporting Information [file CPR-56-e13396-s001.docx]

**Supplemental Figures and Table**


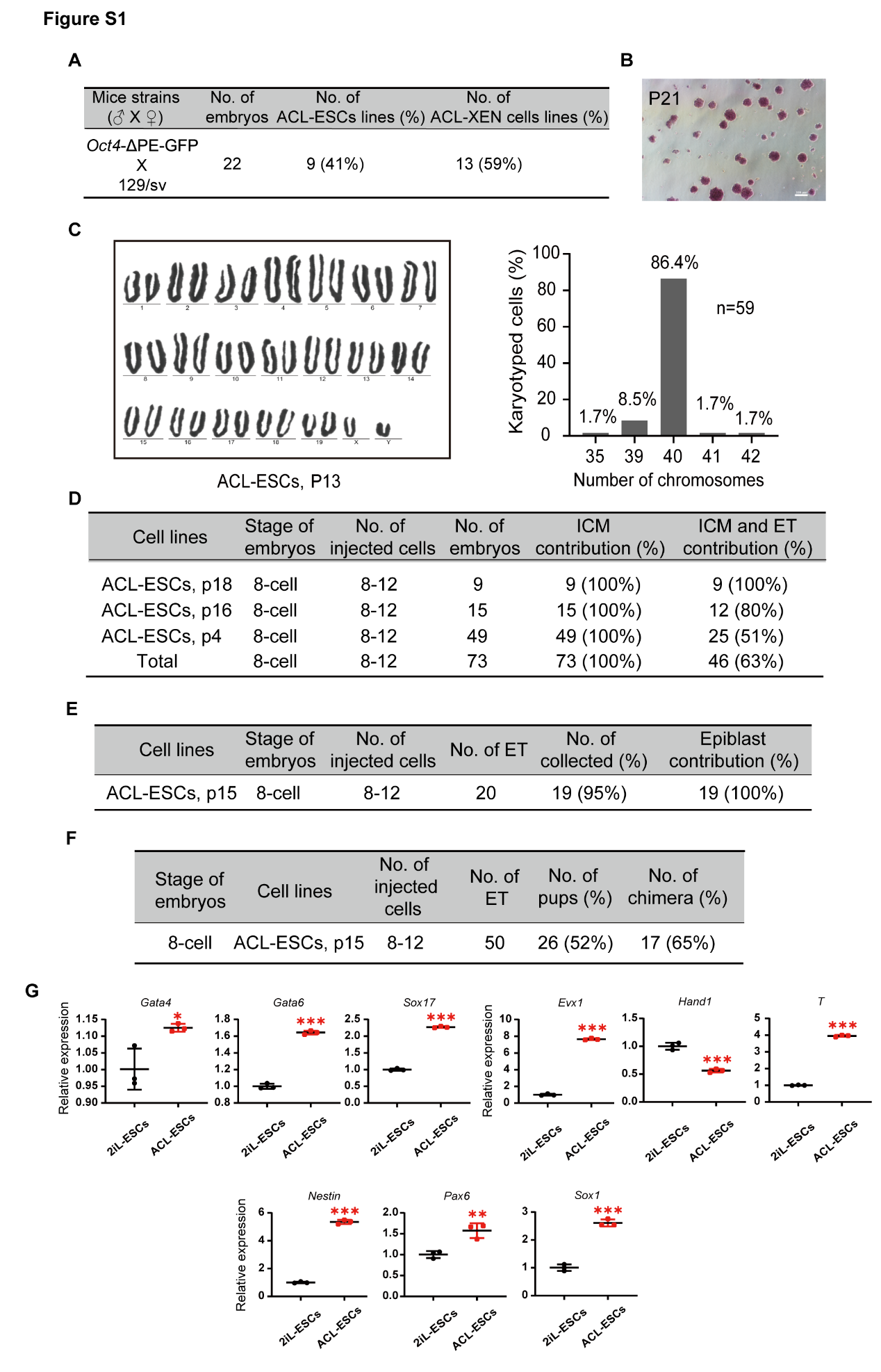


**Figure S1. Karyotyping and Developmental Potency of ACL-ESCs (Related to Figure 1).**

1. Derivation rates of ACL-ESCs and ACL-XEN cells.
2. Alkaline phosphatase (AP) staining of ACL-ESCs. Scale bars, 100 μm.
3. Karyotyping and distribution of chromosome number of ACL-ESCs (P13). Number (59) of spreads analyzed and obtained from 2 independent experiments.
4. Summary of ACL-ESCs contribution to ICM, ICM and TE of chimeras. Chimeras collected after 8-cell embryos injection of ACL-ESCs and cultured for 48h.
5. Summary of ACL-ESCs contribution to epiblast in E6.5 chimeras.
6. Statistics of full-term chimeric pups derived by ACL-ESCs.
7. Relative expression of three germ layers genes measured by qPCR, after ACL-ESCs underwent 3 days for *in vitro* differentiation. Error bars are mean ± SD (n = 3). The *p* values were calculated by two-tailed Student’s *t* test, *p* < 0.05.


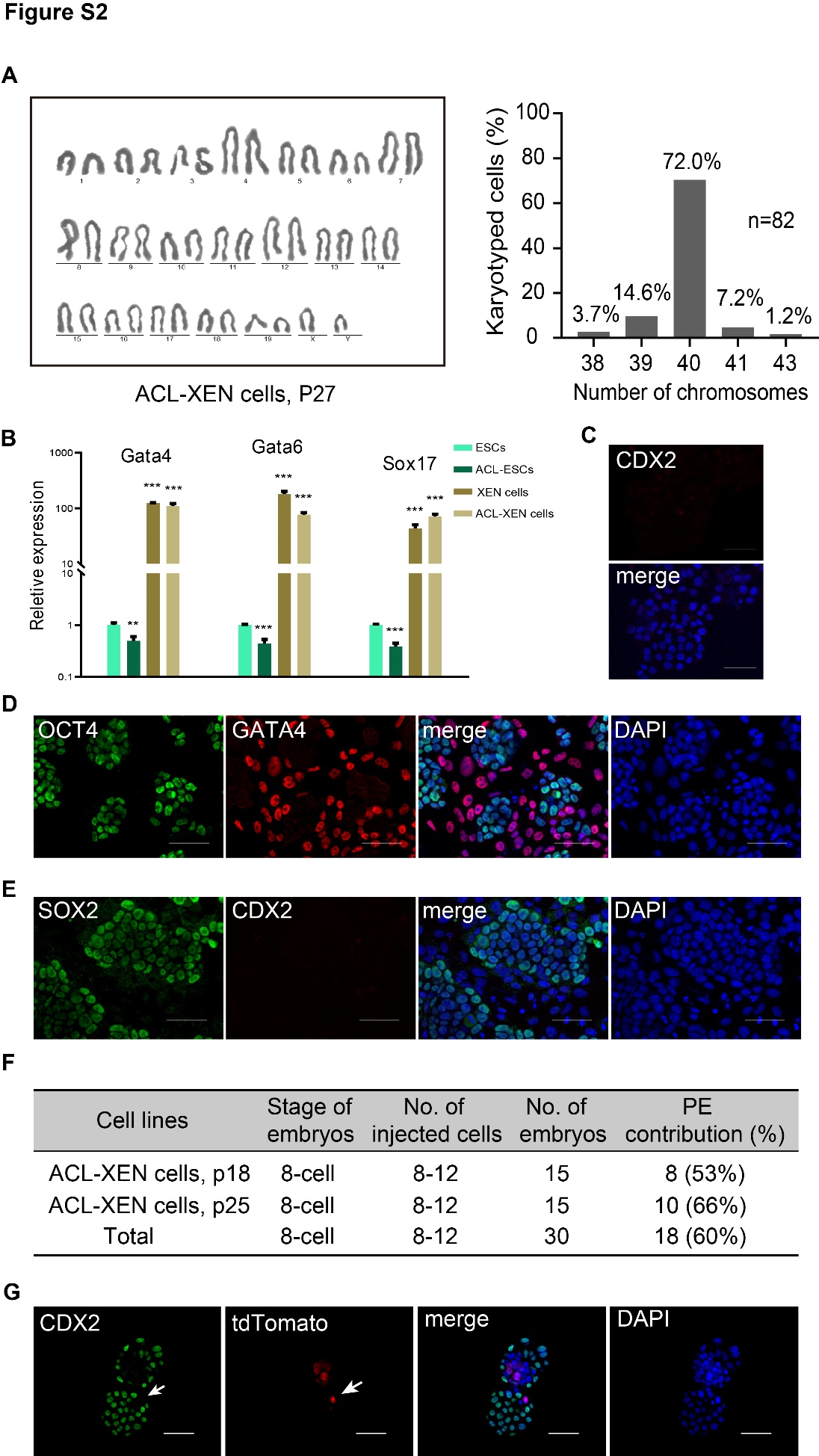


**Figure S2. Characteristics of ACL-XEN cells (Related to Figure 2).**

1. Karyotyping and distribution of chromosome number in ACL-XEN cells. Number (82) of spreads analyzed and obtained from 2 independent experiments.
2. Relative expression of endoderm-associated genes (*Gata4*, *Gata6*, *Sox17*) measured by qPCR in ESCs, ACL-ESCs, XEN cells and ACL-XEN cells. ESCs was used as control. Error bars indicate mean ± SD (n = 3). Results were obtained from three independent experiments. *p* values were calculated by unpaired Student’s *t* test, *p* < 0.05.
3. IF staining assay of CDX2 in ACL-XEN cells. DAPI stained the nucleus. Scale bars, 50 μm.
4. IF staining assays of OCT4 and GATA4 in mixed ACL cells. DAPI stained the nucleus. Scale bars, 50 μm.
5. IF staining assays of SOX2 and CDX2 in mixed ACL cells. DAPI stained the nucleus. Scale bars, 50 μm.
6. Summary of ACL-XEN cells contributed to PrE of chimeras. Chimeras collected after 8-cell embryos injection of ACL-XEN cells and cultured for 48h.
7. IF staining of CDX2 (green) of E4.5 embryos (n=31) generated after 8-cell stage embryos injection of ACL-XEN cells (tdTomato+) and cultured for 48 h *in vitr*o. Arrows indicate ACL-XEN cells with H2B tdTomato+ were no merged with trophectoderm. DAPI stained the nucleus. Scale bars, 50 μm.


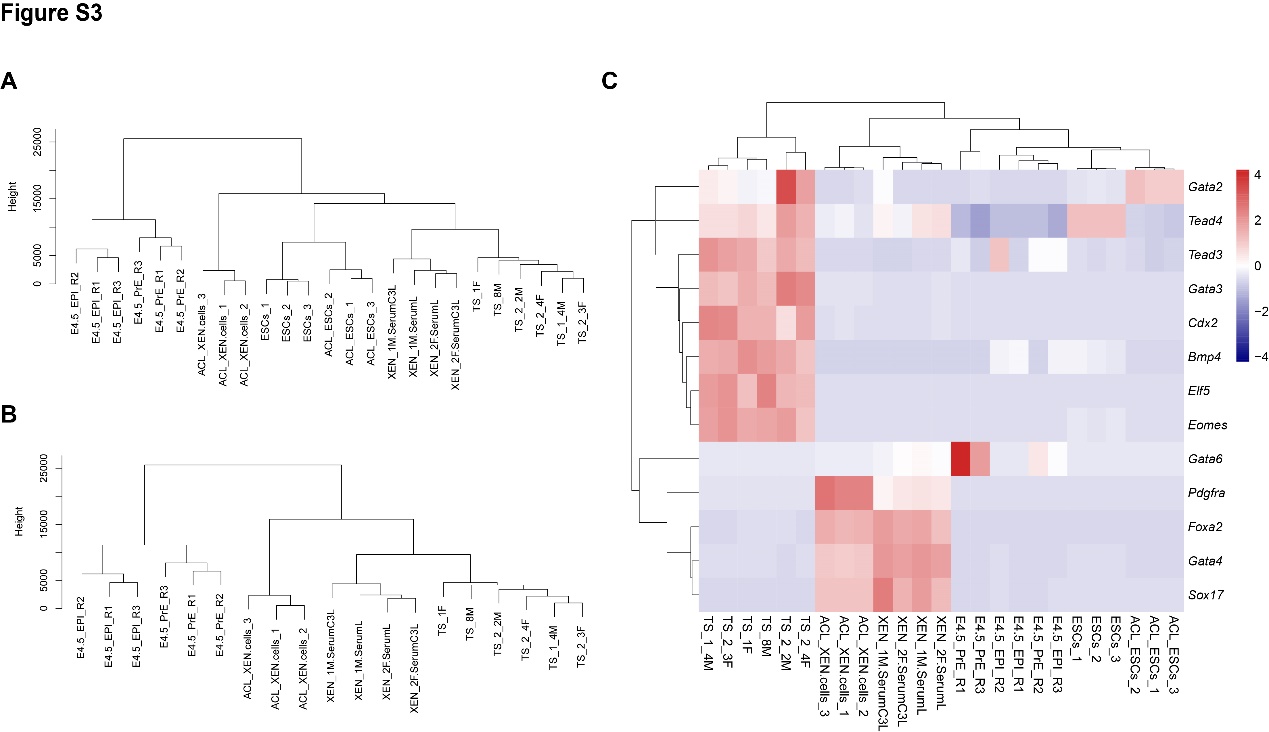


**Figure S3. Transcriptomic characteristics of ACL-ESCs and ACL-XEN cells. (Related to Figure 3).**

1. Unsupervised hierarchical clustering (UHC) of whole-genome transcriptome of ACL-ESCs, ACL-XEN cells, ESCs, TSCs, XEN cells, E4.5_Epi (Epiblast) and E4.5_PrE (primitive endoderm).
2. Unsupervised hierarchical clustering (UHC) of whole-genome transcriptome of ACL-XEN cells, TSCs, XEN cells, E4.5_Epi and E4.5_PrE.
3. Heatmap showing trophectoderm- and endoderm-related genes in ACL-ESCs, ACL-XEN cells, ESCs, TSCs, XEN cells, E4.5_Epi and E4.5_PrE based on RNA-seq data.


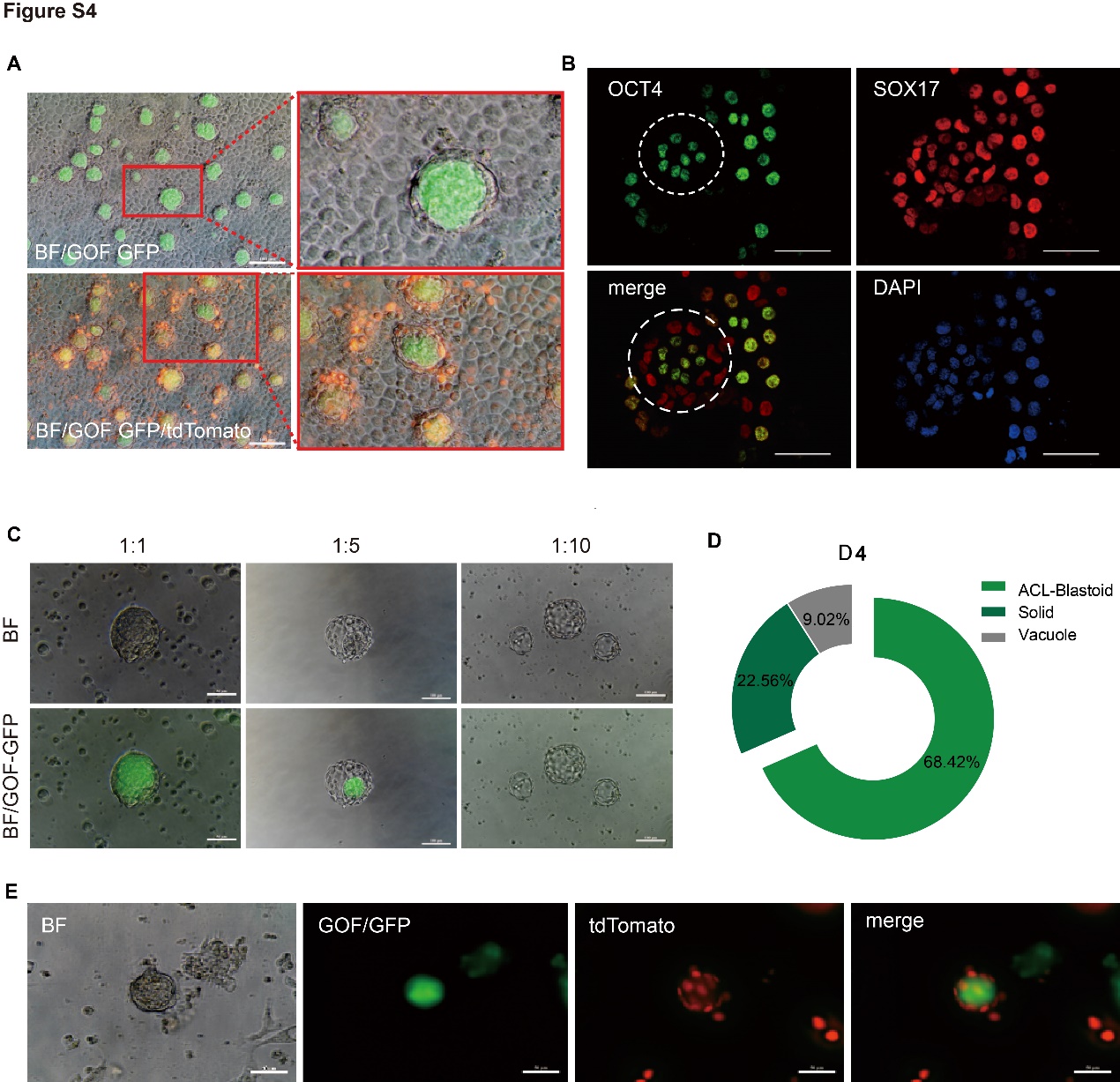


**Figure S4. Process of ACL-blastoid derivation (Related to Figure 4).**

1. The merged live images of 2D culture for 72 h of ACL-ESCs mixed with ACL-XEN cells showed the ACL-ESCs (GOF/GFP+) in the center of colonies (top) and ACL-XEN cells (tdTomato+) surrounding ACL-ESCs (GOF/GFP+) (bottom). DAPI stained the nucleus.Scale bars, 100 μm.
2. Immunofluorescence staining for 2D culture for 72h of ACL-ESCs mixed with ACL-XEN cells indicated that OCT4+ cells were surrounded with SOX17+ cells. DAPI stained the nucleus. Scale bars, 50 μm.
3. The dominating structures of ACL aggregations with the proportion of 1:1, 1:5 and 1:10. Green fluorescence indicated ICM-like cells derived from ACL-ESCs with GOF/GFP in ACL aggregations. BF, bright-field. Scale bars, left: 50 μm, middle and left: 100 μm.
4. Quantification of ACL-blastoids formation efficiency. n = 133 cell aggregates.
5. Aggregation in ETX medium with ACL-ESCs (GOF/GFP) and ACL-XEN cells (tdTomato+). Channels shown separately and merged. BF, bright-field. Scale bars, 50 μm.


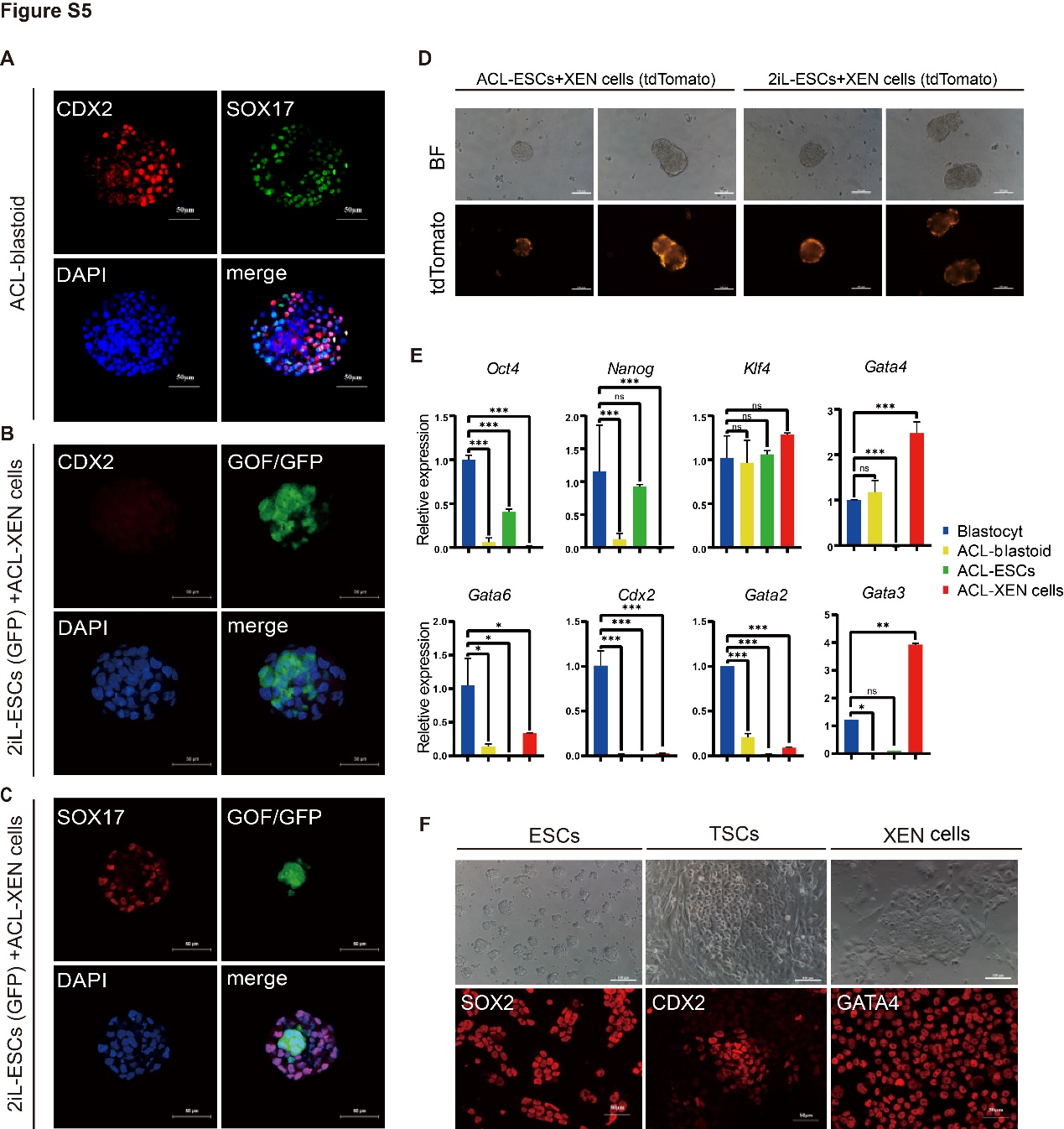


**Figure S5. Characteristics of ACL-blastoid (Related to Figure 5, 6)**

1. IF staining of CDX2 (red) and SOX17 (green) in ACL-blastoids at day 5. Channels shown separately and merged. DAPI stained the nucleus. Scale bars, 50 μm.
2. IF staining of CDX2 (red) aggregates with ESCs (GOF/GFP+) and ACL-XEN cells at day 5. Channels shown separately and merged. DAPI stained the nucleus. Scale bars, 50 μm.
3. IF staining of SOX17 (red) aggregates with ESCs (GOF/GFP+) and ACL-XEN cells at day 5. DAPI stained the nucleus. Scale bars, 50 μm.
4. Bright-field and fluorescent images of XEN cells (tdTomato+) aggregating with ACL-ESCs and ESCs, respectively. BF, bright-field; Scale bars, 100 μm.
5. Relative expression of three blastocyst lineages genes (*Oct4, Nanog, Klf4, Gata4, Gata6, Cdx2, Gata2* and *Gata3*) measured by qPCR in individual blastocyst at day 4, ACL-blastoid at day 5, ACL-ESCs and ACL-XEN cells. Blastocyst was used as control. Error bars indicate mean ± SD (n = 3). Results were obtained from three independent experiments. *p* values were calculated by unpaired two-tailed Student’s *t* test, *p* < 0.05.
6. Bright-field images of ESCs, TSCs and XEN cells lines (top) derived from ACL-blastoids, respectively, staining for SOX2, CDX2 and GATA4 (bottom). Scale bars, top: 100 μm, bottom: 50 μm.

**Supplemental Table S1.** Primer used for qRT-PCR (Related to Figures 5H, S1G, S2B, and S5E)

| **Gene** | **Forward Primer** | **Reverse Primer** |
| --- | --- | --- |
| *Oct3/4* | GATGCTGTGAGCCAAGGCAAG | GGCTCCTGATCAACAGCATCAC |
| *Nanog* | CTTTCACCTATTAAGGTGCTTGC | TGGCATCGGTTCATCATGGTAC |
| *Klf4* | GCACACCTGCGAACTCACAC | GTTTGCGGTAGTGCCTGGTC |
| *Gata4* | TTCCTCTCCCAGGAACATCAAA | GCTGCACAACTGGGCTCTACTT |
| *Gata6* | TGCTGGAAATTGCAACAAACC | GTCACGTGGTACAGGCGTCA |
| *Sox17* | GTCAACGCCTTCCAAGACTTG | GTAAAGGTGAAAGGCGAGGTG |
| *Brachyury* | GAACCTCGGATTCACATCGT | TTCTTTGGCATCAAGGAAGG |
| *Evx1* | CCAGTGACCAGATGCGCCGATAC | TCCTTCATGCGCCGGTTCT |
| *Hand1* | TCAAAAAGACGGATGGTGGT | GCGCCCTTTAATCCTCTTCT |
| *GAPDH* | ATGGTGAAGGTCGGTGTGAAC | TCGCTCCTGGAAGATGGTGATG |
| *Rex1* | CGTGTAACATACACCATCCG | GAAATCCTCTTCCAGAATGG |
| *Gata2* | GATGAATGGACAGAACCGGC | GTGGCCTGTTAACATTGTGC |
| *Gata3* | GCTCCTTGCTACTCAGGTGAT | GGAGGGAGAGAGGAATCCGA |
| *Eomes* | CGGCAAAGCGGACAATAACA | GGAGCCAGTGTTAGGAGATTC |
| *Nestin* | CTCGAGCAGGAAGTGGTAGG | TTGGGACCAGGGACTGTTAG |
| *Sox1* | GGCCGAGTGGAAGGTCATGT | TCCGGGTGTTCCTTCATGTG |
| *Pax6* | GCAGATGCAAAAGTCCAGGTG | CAGGTTGCGAAGAACTCTGTTT |
| Cdx2 | CCTGCGACAAGGGCTTGTTTAG | TCCCGACTTCCCTTCACCATAC |
